# Albumin-assembled copper-bismuth bimetallic sulfide bioactive nanosphere as an amplifier of oxidative stress for enhanced radio-chemodynamic combination therapy

**DOI:** 10.1093/rb/rbac045

**Published:** 2022-07-05

**Authors:** Weiyong Tao, Zhan Tuo, Feige Wu, Ketao Mu, Cunjing Xu, Yuxiao Shi, Zeyu Sun, Yifan Wang, Yan Li, Zhenyu Zhong, Lei Zhou, Jianglin Wang, Jie Liu, Yingying Du, Shengmin Zhang

**Affiliations:** Advanced Biomaterials and Tissue Engineering Center, Huazhong University of Science and Technology, Wuhan 430074, China; NMPA Research Base of Regulatory Science for Medical Devices & Institute of Regulatory Science for Medical Devices, Huazhong University of Science and Technology, Wuhan 430074, China; Department of Biomedical Engineering, Huazhong University of Science and Technology, Wuhan 430074, China; Cancer Center, Union Hospital, Tongji Medical College, Huazhong University of Science and Technology, Wuhan 430022, China; Advanced Biomaterials and Tissue Engineering Center, Huazhong University of Science and Technology, Wuhan 430074, China; NMPA Research Base of Regulatory Science for Medical Devices & Institute of Regulatory Science for Medical Devices, Huazhong University of Science and Technology, Wuhan 430074, China; Department of Biomedical Engineering, Huazhong University of Science and Technology, Wuhan 430074, China; Department of Radiology, Tongji Hospital, Tongji Medical College, Huazhong University of Science and Technology, Wuhan 430030, China; Advanced Biomaterials and Tissue Engineering Center, Huazhong University of Science and Technology, Wuhan 430074, China; NMPA Research Base of Regulatory Science for Medical Devices & Institute of Regulatory Science for Medical Devices, Huazhong University of Science and Technology, Wuhan 430074, China; Department of Biomedical Engineering, Huazhong University of Science and Technology, Wuhan 430074, China; Advanced Biomaterials and Tissue Engineering Center, Huazhong University of Science and Technology, Wuhan 430074, China; NMPA Research Base of Regulatory Science for Medical Devices & Institute of Regulatory Science for Medical Devices, Huazhong University of Science and Technology, Wuhan 430074, China; Department of Biomedical Engineering, Huazhong University of Science and Technology, Wuhan 430074, China; Advanced Biomaterials and Tissue Engineering Center, Huazhong University of Science and Technology, Wuhan 430074, China; NMPA Research Base of Regulatory Science for Medical Devices & Institute of Regulatory Science for Medical Devices, Huazhong University of Science and Technology, Wuhan 430074, China; Department of Biomedical Engineering, Huazhong University of Science and Technology, Wuhan 430074, China; Advanced Biomaterials and Tissue Engineering Center, Huazhong University of Science and Technology, Wuhan 430074, China; NMPA Research Base of Regulatory Science for Medical Devices & Institute of Regulatory Science for Medical Devices, Huazhong University of Science and Technology, Wuhan 430074, China; Department of Biomedical Engineering, Huazhong University of Science and Technology, Wuhan 430074, China; Advanced Biomaterials and Tissue Engineering Center, Huazhong University of Science and Technology, Wuhan 430074, China; NMPA Research Base of Regulatory Science for Medical Devices & Institute of Regulatory Science for Medical Devices, Huazhong University of Science and Technology, Wuhan 430074, China; Department of Biomedical Engineering, Huazhong University of Science and Technology, Wuhan 430074, China; Advanced Biomaterials and Tissue Engineering Center, Huazhong University of Science and Technology, Wuhan 430074, China; NMPA Research Base of Regulatory Science for Medical Devices & Institute of Regulatory Science for Medical Devices, Huazhong University of Science and Technology, Wuhan 430074, China; Department of Biomedical Engineering, Huazhong University of Science and Technology, Wuhan 430074, China; Advanced Biomaterials and Tissue Engineering Center, Huazhong University of Science and Technology, Wuhan 430074, China; NMPA Research Base of Regulatory Science for Medical Devices & Institute of Regulatory Science for Medical Devices, Huazhong University of Science and Technology, Wuhan 430074, China; Department of Biomedical Engineering, Huazhong University of Science and Technology, Wuhan 430074, China; Advanced Biomaterials and Tissue Engineering Center, Huazhong University of Science and Technology, Wuhan 430074, China; NMPA Research Base of Regulatory Science for Medical Devices & Institute of Regulatory Science for Medical Devices, Huazhong University of Science and Technology, Wuhan 430074, China; Department of Biomedical Engineering, Huazhong University of Science and Technology, Wuhan 430074, China; School of Biomedical Engineering, Sun Yat-sen University, Guangzhou, Guangdong 510006, China; Advanced Biomaterials and Tissue Engineering Center, Huazhong University of Science and Technology, Wuhan 430074, China; NMPA Research Base of Regulatory Science for Medical Devices & Institute of Regulatory Science for Medical Devices, Huazhong University of Science and Technology, Wuhan 430074, China; Department of Biomedical Engineering, Huazhong University of Science and Technology, Wuhan 430074, China; Advanced Biomaterials and Tissue Engineering Center, Huazhong University of Science and Technology, Wuhan 430074, China; NMPA Research Base of Regulatory Science for Medical Devices & Institute of Regulatory Science for Medical Devices, Huazhong University of Science and Technology, Wuhan 430074, China; Department of Biomedical Engineering, Huazhong University of Science and Technology, Wuhan 430074, China

**Keywords:** bioactive materials, nanosphere, assembly, bismuth–copper, radio-chemodynamic therapy

## Abstract

The tumor microenvironment with overexpressed hydrogen peroxide (H_2_O_2_) and reinforced antioxidative system (glutathione, GSH) becomes a double-edged sword for the accessibility of nano-therapy. Since reactive oxygen species (ROS) are easily quenched by the developed antioxidative network, ROS-based treatments such as chemodynamic therapy (CDT) and radiotherapy (RT) for killing cancer cells are severely attenuated. To overcome such limitations, a bioactive nanosphere system is developed to regulate intracellular oxidative stress for enhanced radio-chemodynamic combination therapy by using bovine serum albumin (BSA) based bioactive nanospheres that are BSA assembled with *in situ* generated copper-bismuth sulfide nanodots and diallyl trisulfide (DATS). The copper-bismuth sulfide nanodots react with H_2_O_2_ to produce •OH and release Cu^2+^. Then, the Cu^2+^ further depletes GSH to generate Cu^+^ for more •OH generation in the way of Fenton-like reaction. Such a cascade reaction can initiate •OH generation and GSH consumption to realize CDT. The elevation of ROS triggered by the DATS from BBCD nanospheres further augments the breaking of redox balance for the increased oxidative stress in 4T1 cells. With the sensitization of increased oxidative stress and high Z element Bi, an enhanced radio-chemodynamic combination therapy is achieved. The current work provides an enhanced radio-chemodynamic combination treatment for the majority of solid tumors by using the co-assembled bioactive nanospheres as an amplifier of oxidative stress.

## Graphical Abstract

**Figure:**
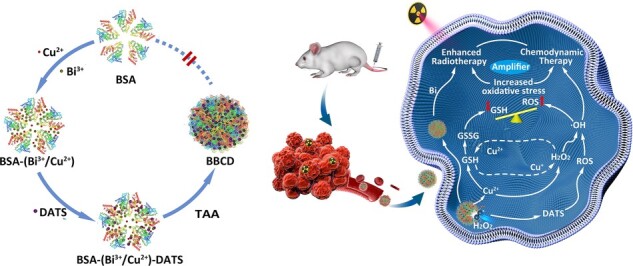

## Introduction

Intracellular redox status is maintained by dynamic equilibrium between reactive oxygen species (ROS) and the antioxidative defense system. ROS, such as hydroxyl radical (•OH), superoxide anion ($O_{2}^{•-}$), singlet oxygen (^1^O_2_) and others, can cause cell death by oxidizing biological matter in cells [1–3]. The antioxidative system in cancer cells consists of multiple small molecules, antioxidant enzymes and other specific components [4–6]. As a ROS capturer, glutathione (GSH) is an important member in the antioxidative system that can neutralize ROS to protect cells from oxidative damage [7, 8]. The majority of solid tumors have a unique microenvironment with overexpressed GSH (≈10 mM) [9] and H_2_O_2_ (10–50 μM) [10]. This featured tumor microenvironment is an obstacle but also an opportunity for tumor therapy. The inadequate ROS generated from H_2_O_2_ in most solid tumor cells is still difficult to meet the requirements of high therapeutic efficiency [11]. So, a variety of emerging materials have been designed for the enhanced level of H_2_O_2_ or ROS in cancer cells [12–18]. For example, Koo *et al*. [19] synthesized the copper–iron peroxide nanoparticles for the supply of H_2_O_2_, and the Cu and Fe ions synergistically initiated the production of abundant ROS by the Fenton reaction. However, the reinforced antioxidant system in tumor cells can eliminate ROS before their interaction with intracellular components to attenuate the outcome of ROS therapy [20]. So, a single nanosystem with enhanced ROS generation as well as GSH depletion is urgently needed for cancer therapy.

Radiotherapy (RT) is one of the most widely used treatments in clinic [21]. Great effort has been dedicated to meliorating the efficiency of RT [22, 23], for instance, high Z elements for X-ray deposition [24–26] and delivering oxygen for reversing hypoxia [27, 28]. However, the abnormal metabolism in tumor tissues triggers the mutation of redox homeostasis [29]. Oxidative damages (DNA double-strand destruction or lipid peroxidation) initiated by ionizing radiation (e.g. X-ray) are more easily repaired by the reinforced antioxidant system in cancer cells, resulting in a restricted RT [30, 31]. Therefore, disrupting the intra-tumoral redox balance for enhanced oxidative damage caused by ionizing radiation is a promising way of sensitization for RT.

**Figure 1.**
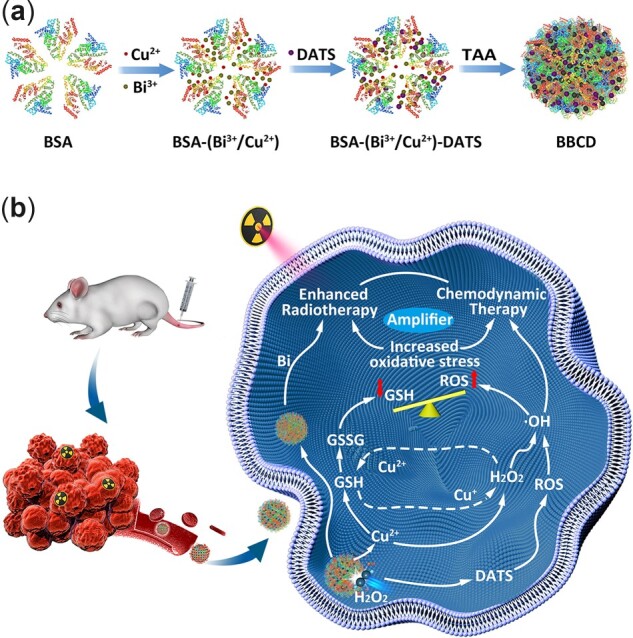
The synthesis route and therapy mechanism of BBCD. (**a**) The fabrication procedure of BBCD nanospheres. (**b**) The mechanism of BBCD for enhanced radio-chemodynamic combination therapy.

Based on the predicament mentioned above, BSA-based copper-bismuth sulfide/DATS nanospheres (BBCD) are designed to modulate redox balance, constructing an internal microenvironment with a high level of oxidative stress in the tumor for amplified radio-chemodynamic combination therapy. As depicted in Fig. 1a, an integrated nanoplatform of BBCD is fabricated facilely through an *in situ* assembly route at room temperature. Bi^3+^ and Cu^2+^ chelated bovine serum albumin (BSA) assembles with diallyl trisulfide (DATS), thioacetamide (TAA) acts as the donor of S^2^^−^ to induce ultrasmall copper-bismuth sulfide (BC) nanodots growing inside organic molecules, BBCD nanospheres are obtained after aging. Nanospheres of BBC (BC nanodots loaded in BSA nanosphere) or BB (bismuth sulfide nanodots loaded in BSA nanosphere) are synthesized as control samples. The enhanced therapeutic efficacy by BBCD is illustrated in Fig. 1b. (i) Consumption of GSH for enhanced chemodynamic therapy (CDT). BBCD nanospheres interact with intratumoral GSH and H_2_O_2_ to generate •OH and deplete GSH in the way of Fenton-like reaction. The GSH depletion leads to enhanced CDT. (ii) Increased oxidative stress. DATS can induce an enhanced level of ROS in 4T1 cells, and the elevated ROS and decreased GSH lead to amplified oxidative stress. (iii) Enhanced RT. The amplification of oxidative stress within tumor cells reverses the resistance induced by the antioxidative system, making 4T1 cells more sensitive to oxidative damage induced by RT. Thus, an amplified RT is achieved. In a word, BBCD nanospheres are successfully fabricated to prove an effective regulation of oxidative stress in 4T1 cells for elevated radio-chemodynamic combination therapy, providing a potential strategy for the sensitization of ROS-based tumor therapy.

## Materials and methods

### Materials

*Materials*. Bismuth nitrate pentahydrate (Bi(NO_3_)_3_·5H_2_O, 99%), copper nitrate trihydrate (Cu(NO_3_)_2_·3H_2_O, 99%), TAA, dimethyl sulfoxide (DMSO), nitric acid (HNO_3_), methylene blue (MB, ≥82%) were purchased from Sinopharm Chemical Reagent Co., Ltd (China). DATS (≥98%) was bought from Beijing Jin Ming Biotechnology Co., Ltd. BSA, GSH, 3,3′,5,5′-tetramethylbenzidine (TMB), terephthalic acid (TPA) and 5,5′-dithiobis-(2-nitrobenzoic acid) (DTNB) were obtained from Sigma-Aldrich. No further purification for all used reagents were operated. The catalog numbers of reagents: Bi(NO_3_)_3_·5H_2_O (Sinopharm Chemical Reagent Co., Ltd, catalog number: 80018318), Cu(NO_3_)_2_·3H_2_O (Sinopharm Chemical Reagent Co., Ltd, catalog number: 10007916), TAA (Sinopharm Chemical Reagent Co., Ltd, catalog number: 30177714), DMSO (Sinopharm Chemical Reagent Co., Ltd, catalog number: 30072428), DATS (Beijing Jin Ming Biotechnology Co., Ltd, catalog number: D60006), nitric acid (Sinopharm Chemical Reagent Co., Ltd, catalog number: 10014528), BSA (Sigma-Aldrich, B265993-25g), MB (Sinopharm Chemical Reagent Co., Ltd, catalog numbers: 71024544), GSH (Sigma-Aldrich, G105427-5g), 3,3′,5,5′-tetramethylbenzidine (Sigma-Aldrich, T100417), TPA (Sigma-Aldrich, P108506-100g), 5,5′-dithiobis-(2-nitrobenzoic acid) (Sigma-Aldrich, D105559).

### Synthesis and characterization

#### Synthesis of BBCD

BBCD nanospheres were synthesized through a modified method based on the previous report [32]. Forty-eight milligrams of Bi(NO_3_)_3_·5H_2_O were dissolved in 2 ml HNO_3_ (1.5 M) solution, 12 mg Cu(NO_3_)_2_·3H_2_O were dissolved in 2 ml deionized H_2_O and 15 mg BSA were dissolved in 20 ml deionized water. Then, the solution of Bi^3+^ and Cu^2+^ were dropwise added into BSA solution, respectively. After 30 min stirring, 20 mg DATS in 200 μl DMSO were injected into above mixture. Thirty minutes later, 2 ml TAA solution (0.004 g/ml) was added slowly for another 6 h stirring at 25°C. Then, BBCD nanospheres were obtained by centrifugation at 12 000 rpm for 10 min. BBC nanospheres were synthesized in the same procedure described above without the addition of DATS. BB nanospheres were synthesized in the same route depicted above without addition of Cu(NO_3_)_2_·3H_2_O and DATS.

#### Characterization

Transmission electron microscopy (TEM) images and high-resolution TEM (HRTEM) images were captured by Talos F200X electron microscope. Scanning electron microscopy (SEM) images were taken by Nova NanoSEM 450 electron microscope. Energy dispersive spectrometry (EDS) mapping (Talos F200X) and X-ray fluorescence (XRF) (EAGLE III) were used to analyze the composition of elements in nanospheres. The X-ray diffraction (XRD) analysis of samples were operated on X’Pert3 Powder. X-ray photoelectron spectroscopy (XPS) analysis for the valence state of elements in samples were conducted on machine of AXIS-ULTRA DLD-600W. Inductively coupled plasma-mass spectrometry (ICP-MS) analysis for the content of elements in samples was operated on PerkinElmer ELAN DRC-e. The level of DATS in BBCD was determined by thermogravimetric (TG) analyzer (Diamond TG/DTA, PerkinElmer), with an increasing rate of 20°C/min from room temperature to 650°C under air atmosphere. Fourier transform infrared spectroscopy (FT-IR) and UV–vis absorption of the samples were recorded through Nicolet iS50R (Thermo Scientific) and SolidSpec-3700 (Shimadzu) spectrophotometer. FP-6500 (Jasco) spectrometer was used to detect the fluorescence spectra. Fluorescence microscope (Nikon Ti2-U) and flow cytometer (CytoFLEX, Beckman) were used for the fluorometric analysis.

### Reaction of BBCD with H_2_O_2_ or GSH

#### Reaction between BBCD and H_2_O_2_

BBCD (100 µg/ml) reacted with H_2_O_2_ (10 mM) in PBS solution at 37°C for 12 h. The precipitations obtained by centrifugation at different times (30 min, 1, 2 and 12 h) were characterized by TEM. After 12 h reaction, the precipitates (BBCD_H_) were collected for the analysis of XPS, XRD and XRF.

•OH production from Cu^2+^ reacted with H_2_O_2_:TMB (50 μg/ml) incubated with Cu^2+^ (1 mM) and H_2_O_2_ (1 mM or 10 mM) in PBS solution at 37°C. BBCD (100 μg/ml) reacted with H_2_O_2_ (10 mM) in a PBS solution containing TMB (50 μg/ml) at 37°C. The system of TMB, TMB + Cu^2+^, TMB + H_2_O_2_ were set as the control groups. After 2 h reaction, all systems were centrifuged at 10 000 rpm for the detection of absorption at 650 nm via UV–vis spectroscopy.

#### Degradation of MB and TPA oxidation

MB (20 μg/ml) and (i) BBCD (100 µg/ml), (ii) BBC (88 µg/ml) and (iii) BB (91 µg/ml) were treated with H_2_O_2_ (10 mM) in PBS solution, the degradation of MB induced by •OH at different time were measured through UV–vis spectroscopy. The persistently generation of •OH was measured via TPA oxidation at various concentrations of H_2_O_2_. PBS solutions including TPA (1 mg/ml), BBCD (100 µg/ml) and different concentration of H_2_O_2_ (0, 0.5, 1, 5 and 10 mM) were stirred at 37°C for 2 h, then the fluorescence intensity induced by excitation wavelength at 315 nm was measured at around 430 nm.

#### Reaction of BBCD with GSH

BBCD (1 mg/ml) reacted with GSH (1 mM) in PBS solution at 37°C for 12 h. Then, the precipitates (BBCD_G_) were obtained by centrifugation for the XPS analysis.

#### Depletion of GSH in solution

The reaction between GSH and DTNB was used to measure the GSH depletion in solution. GSH (1 mM), H_2_O_2_ (0 µM or 500 µM) and (i) no additive as control, (ii) BB (0.91 mg/ml), (iii) BBC (0.88 mg/ml) or (iv) BBCD (1 mg/ml) in PBS were stirred at 37°C for 12 h, and then centrifuged (10 000 rpm) to remove precipitates, The supernatant was incubated with DTNB solution (5 mg/ml, 10 µl) in 96-well plates (*n* = 3) for measurement of absorbance at 412 nm on a microplate reader (Biotech ELx808, USA).

### Cell culture

The 4T1 cancer cells were purchased from Wuhan Kehaojia Biotechnology Co., LTD. Mouse bone marrow stromal cells (BMSC) and human umbilical vein vessel endothelial cells (HUVECs) were obtained from Advanced Biomaterials and Tissue Engineering Center, HUST. All the cells were cultured with DMEM (including 10% fetal bovine serum) in a humidified 5% CO_2_ incubator at 37°C.

### Analysis of intracellular ROS and GSH/GSSG

#### Evaluation of intracellular ROS

4T1 cells were seeded into 6-well plates with a density of 1 × 10^6^ cells per well. After 12 h incubation, the culture medium was replaced with fresh medium, which included: (i) no additive as control, (ii) BB (91 µg/ml), (iii) BBC (88 µg/ml), (iv) BBCD (100 µg/ml). After 18 h co-culturing, the treated cells were washed by PBS and incubated with 2′,7′-dichlorodihydrofluorescein diacetate (DCFH-DA, 10 μM in pure DMEM) for 30 min at 37°C in dark. The stained cells were analyzed by fluorescence microscope and flow cytometer.

4T1 cells were pretreated by BB (91 µg/ml), BBC (88 µg/ml) or BBCD (100 µg/ml) for 12 h, then exposed to X-ray irradiation (6 Gy). After another 6 h culturing, the treated cells were washed by PBS and stained by DCFH-DA (10 μM). ROS in treated cells were tested by fluorescence microscope and flow cytometer.

4T1 cells were pretreated by BB (91 µg/ml) + GSH (500 μM), BBC (88 µg/ml) + GSH (500 μM) or BBCD (100 µg/ml) + GSH (500 μM) for 12 h, then exposed to X-ray irradiation (6 Gy). The ROS level in treated cells were tested by fluorescence microscope after another 6 h incubation.

#### Detection of intracellular GSH

The GSH/GSSG assay kit (Beyotime, Jiangsu, China) was used to detect intracellular GSH and GSSG. 4T1 cells were cultured in six-well microplates (1 × 10^6^ cells per well) for 12 h. Then the medium was replaced by fresh medium, which contained: (i) no additive as control, (ii) BB (91 µg/ml), (iii) BBC (88 µg/ml), (iv) BBCD (100 µg/ml). After 12 h incubation, the treated cells were washed by PBS and collected. Solution of protein removal reagent was mixed with the treated cells for rapid vortex, and then the samples were subjected to two rapid freeze–thaw cycles in liquid nitrogen and 37°C water bath. The supernatant was obtained after centrifugation of 10 000 g for 10 min. Detection of GSH and GSSG in samples was performed according to the protocol in GSH/GSSG assay kit.

### Western blotting assay and RNA-sequencing

4T1 cells were treated in the same way as the experiment of GSH detection. The treated cells were mixed with appropriate amount of RIPA lysate containing 1 mmol/L phenylmethanesulfonyl fluoride and phosphatase inhibitors (Servicebio, Wuhan) for 30 min incubation. The samples were centrifugated (12 000 rpm, 30 min at 4°C) to collect the supernatant. The protein samples were subjected to 10% polyacrylamide SDS-PAGE. Then the primary antibodies anti-HMOX1 (1:1000, Abclonal, A19162) and anti-GAPDH (1:4000, Abclonal, AC002) were used for the analysis of immunoblots. After incubation with the goat anti-rabbit IgG secondary antibody, signals of protein were recorded.

4T1 cells were seeded in 6-well plates with a density of 1 × 10^6^ cells per well for 24 h incubation. The 4T1 cells incubated with BBCD (100 µg/ml) for 12 h. Then the treated 4T1 cells were collected to perform the high-throughput sequencing (Wuhan SeqHealth Tech Co., Ltd).

### Tumor killing *in vitro*

#### Cytotoxicity in vitro

The cell viability was measured by CCK-8 (Cell Counting Kit-8, US EVERBRIGHT INC.). 4T1 cells were seeded in 96-well microplates (6 × 10^3^ cells per well) for 24 h incubation. The 4T1 cells were treated with BB, BBC or BBCD at different concentration. After another 36 h incubation, the cell viability was detected by microplate reader. 4T1 cells were pretreated by different concentration of BBCD (BB, BBC) for 12 h, then exposed to X-ray irradiation (6 Gy). After another 24 h incubation, the viability of 4T1 cells was tested.

#### Calcein-AM and PI staining

1 × 10^6^ 4T1 cells per well were cultured in six-well plates at 37°C for 24 h. Then the 4T1 cells were treated in the same way as the experiment of cytotoxicity *in vitro*. The groups were named as: (i) no treating as control, (ii) BB (91 µg/ml), (iii) BBC (88 µg/ml), (iv) BBCD (100 µg/ml), (v) RT, (vi) BB (91 µg/ml) + RT, (vii) BBC (88 µg/ml) + RT, (viii) BBCD (100 µg/ml) + RT. Finally, the treated 4T1 cells were washed with PBS and stained by Calcein-AM (10 μM) and PI (20 μM) (US EVERBRIGHT INC.).

**Detection of DNA damage in treated 4T1 cells**

γ-H_2_AX immunofluorescent staining were used to detect the breakage of DNA double-strand in treated 4T1 cells. 4T1 cells in six-well plates were co-cultured with BB (91 µg/ml), BBC (88 µg/ml) or BBCD (100 µg/ml) for 12 h, then exposed to X-ray irradiation (0 or 2 Gy). After 24 h incubation, all groups were washed with PBS for staining of γ-H_2_AX immunofluorescent. The images of immunofluorescence were obtained through fluorescence microscope.

### Tumor therapy *in vivo*

#### Animals

Balb/c mice were obtained from the Laboratory Animal Center, Three Gorges University (Yichang, China). The animal experiments were approved by the Animal Experiment Ethics Committee of Huazhong University of Science and Technology.

#### Tumor models

Balb/c mice (female, 6 weeks) were subcutaneously injected with 100 µl PBS containing ≈10^6^ 4T1 cells. When the tumor volume reached ≈60 mm^3^, the Balb/c mice were randomly divided into eight groups (*n* = 3): (i) PBS, (ii) BB, (iii) BBC, (iv) BBCD, (v) PBS + RT, (vi) BB + RT, (vii) BBC + RT, (viii) BBCD + RT. All mice received tail intravenous injection, and the injection dosage was based on the Bi content ([Bi] = 10 mg/kg). Eighteen hours after injection, the pretreated mice were exposed to X-ray irradiation (0 or 6 Gy). The same treatments were performed at Day 1, 4 and 7. During the period of 21 days treatment, the tumor volume and body weight of all mice were recorded every 3 days. The tumor volume was calculated by the equation of *V* = (*LW*^2^)/2, the letter *L*, *W* and *V* were representative of the length, width and volume of tumor, respectively. At Day 22, the tumors of all mice were stripped for hematoxylin and eosin (H&E) staining, terminal deoxynucleotidyl transferase dUTP nick end labeling (TUNEL) staining, γ-H_2_AX immunofluorescence staining.

### Toxicity of BBCD to normal cells and tissues

#### Toxicity in vitro

CCK-8 assay for cell viability of BMSC and HUVECs *in vitro* were performed to detect the acute cytotoxicity induced by BBCD. BMSC or HUVECs were seeded in 96-well microplates. When the cells density reached 80% of the well area, the cells treated with different concentration of BBCD for 24 h. Then, the cell viability was determined according to the absorbance at 450 nm on microplate reader.

#### Toxicity in vivo

Biochemical analysis of blood and histological analysis of major organs were performed to evaluate the long-term toxicity of BBCD *in vivo*. Healthy Balb/c mice (female, 6 weeks) were randomly divided into 3 groups (*n* = 3). (i) Control (healthy mice), (ii) 1 day (1 day after tail intravenous injection of BBCD [Bi] = 10 mg/kg), (iii) 21 days (21 days after tail intravenous injection of BBCD [Bi] = 10 mg/kg). The fresh blood from all mice were extracted for biochemistry analysis, and the major organs (heart, liver, spleen, lung and kidney) were collected for the staining of H&E.

### Statistical analysis

All data were expressed in the way of mean ± standard deviation (SD). The analysis of statistical difference was operated through one-way analysis of variance (ANOVA).

## Results and discussion

### Synthesis and characterization of BBCD

**Figure 2.**
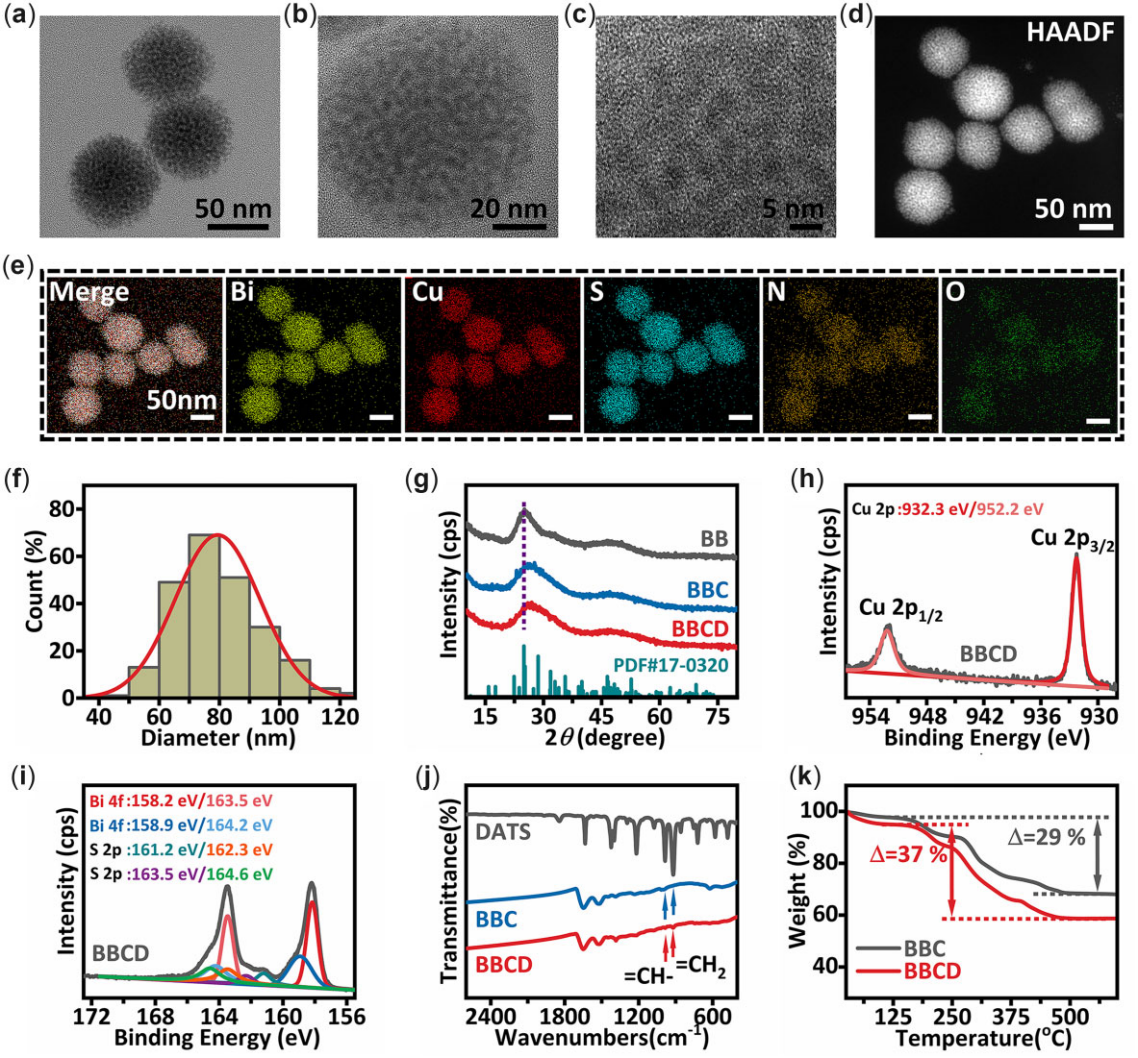
Characterizations of BBCD nanospheres. (**a**, **b**) TEM images of BBCD nanospheres. (**c**) HRTEM image of BBCD nanosphere. (**d**) High-angle annular dark-field (HAADF) image of BBCD nanosphere. (**e**) EDS mappings of elements in BBCD nanosphere, BBCD nanospheres were dispersed on a nickel microgrid. (**f**) The particle size distribution of BBCD nanospheres. (**g**) XRD patterns of BBCD, BBC and BB. (**h**) High-resolution XPS spectra of Cu 2p in BBCD. (**i**) High-resolution XPS spectra of Bi 4f and S 2p in BBCD. (**j**) FT-IR spectra of BBCD, BBC and DATS. (**k**) Thermogravimetric (TG) analysis of BBCD and BBC.

From the images of TEM and SEM (Fig. 2a;Supplementary Fig. S1a–d), monodisperse BBCD spheres with relatively uniform size were obtained. There were about 5 nm nanodots embedded in the organic matrix (Fig. 2b), and no lattice fringes were found by the characterization of HRTEM (Fig. 2c). The images of EDS elemental mapping depicted the elements distribution in the BBCD nanosphere (Fig. 2d and e). As expected, bismuth (Bi), copper (Cu), sulfur (S), nitrogen (N) and trace oxygen (O) elements were throughout the entire spheres with an even distribution. The BBCD spheres have an average size of 80 nm and a larger average hydrodynamic size of 115 nm in PBS (Fig. 2f;Supplementary Fig. S1e). BBC and BB shared the similar characteristics with BBCD (Supplementary Fig. S2 and S3a).

The results of XRD analysis for BBC, BBC and BB were shown in Fig. 2g. The two broad peaks in XRD pattern of BB were indexed to the standard card of Bi_2_S_3_ (JCPDS Card No. 17-0320). Combining the HRTEM and XRD results of BB (Supplementary Fig. S3b), it was concluded that nanodots in BB were bismuth sulfide with poor crystallinity or non-crystallinity [33]. With the addition of Cu, the first diffraction peak in the XRD pattern of BBCD or BBC shifted to a higher diffraction angle, indicating the doping of Cu in bismuth sulfide [34]. XPS analysis was performed to elucidate the valence state of elements in the BBCD nanosphere (Supplementary Fig. S4). The binding energies at 932.3 and 952.2 eV were ascribed to Cu 2p_3/2_ and Cu 2p_1/2_ (Fig. 2h), which corresponded to the standard values of Cu^2+^ in Cu–S bond [35]. There was no difference between the binding energies of Cu 2p in BBCD and BBC (Supplementary Fig. S5). The asymmetric peaks of Bi 4f in the BBCD nanosphere were deconvoluted into four peaks (Fig. 2i). The binding energies at 158.2 and 163.5 eV were attributed to Bi^3+^ in Bi–S. The peaks located at 158.9 and 164.2 eV were assigned to Bi^3+^ in Bi–O, which came from the oxidation of Bi–S at the surface of nanodots [36]. The peaks presented at 161.2 and 162.3 eV were ascribed to the binding energies of S^2^^−^ in BBCD [37], the binding energies at 163.3 and 164.4 eV were identified as S–S in DATS [38, 39]. Based on these results from HRTEM, XRD and XPS analysis, the nanodots in BBCD or BBC were poorly crystalline or amorphous copper-bismuth sulfide. And ICP–MS analysis of BBCD or BBC confirmed that the molar ratio of Bi and Cu in BBCD was not distinctly changing compared with BBC (molar ratio of Bi:Cu_(BBCD)_ = 2.88:1 and Bi:Cu_(BBC)_ = 2.73:1, there were no significant difference between the molar ratio of Bi:Cu_(BBCD)_ and Bi:Cu_(BBC)_, *n* = 3). The FT-IR spectra of BBCD, BBC and DATS were presented in Fig. 2j. The characteristic peaks at 985 and 919 cm^−1^ belonged to the –CH= and =CH_2_ groups in DATS [40, 41], which were only found in the FT-IR spectra of BBCD or DATS, suggesting the successful loading of DATS in BBCD. There were 8 wt.% DATS in BBCD, 61 wt.% copper-bismuth sulfide in BBCD, 69 wt.% copper-bismuth sulfide in BBC and 60 wt.% bismuth sulfide in BB, respectively (Fig. 2k;Supplementary Fig. S7).

### Mechanism of •OH generation and GSH elimination

Based on the findings of TG and ICP-MS analysis, the concentrations of BBCD, BBC and BB used for all the experiments were in corresponding relationships, which guaranteed the level of Bi or Cu in used BBCD, BBC and BB was the same.

**Figure 3.**
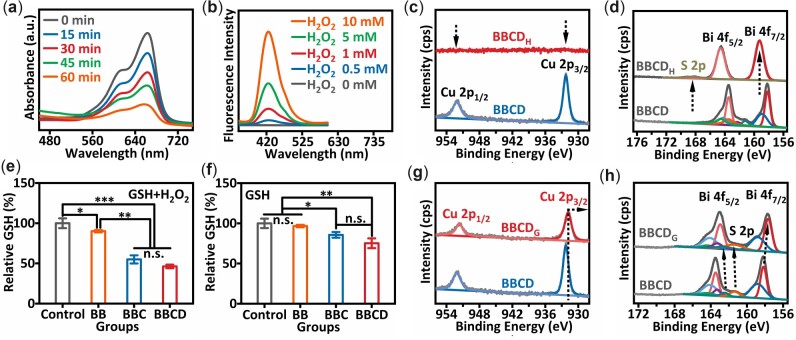
(**a**) UV−vis absorption spectra for the degradation of MB, which was incubated with BBCD and H_2_O_2_. [BBCD] = 100 µg/ml, [H_2_O_2_] = 10 mM, [MB] = 20 µg/ml. (**b**) Fluorescence spectra of TPA oxidized by •OH generated from H_2_O_2_ reacting with BBCD. [BBCD] = 100 µg/ml, [TPA] = 1 mg/ml. (**c**, **d**) High-resolution XPS spectra of Cu 2p, Bi 4f and S 2p in BBCD_H_. (**e**) GSH depleted by BBCD, BBC and BB. GSH as the control group. [BBCD] = 1 mg/ml, [GSH] = 1 mM. (**f**) GSH depleted by BBCD, BBC or BB with addition of H_2_O_2_. The control group was GSH + H_2_O_2_. [BBCD] = 1 mg/ml, [GSH] = 1 mM, [H_2_O_2_] = 500 µM. (**g**, **h**) High-resolution XPS spectra of Cu 2p, Bi 4f and S 2p in BBCD_G_ (mean ± SD, non-significance was expressed as n.s., and *P <* *0.05, **P <* *0.01, ***P <* *0.001, *n* = 3).

As we known, Cu^+^ reacted with H_2_O_2_ to produce •OH in the way of Fenton-like reaction. Cu^2+^ could react with H_2_O_2_ to produce •OH too [42]. The chromogenic products with absorbance at around 650 nm generated from the oxidation of 3,3′,5,5′-tetramethylbenzidine (TMB) was applied to confirm the production of •OH [43]. Firstly, the generated •OH from reaction between Cu^2+^ and H_2_O_2_ was confirmed by the elevated intensity of absorption at around 650 nm (Supplementary Fig. S8). Next, the •OH generation from BBCD reacting with H_2_O_2_ was demonstrated by the oxidation of TMB. Addition of BBCD into the solution including TMB and H_2_O_2_ contributed to an obvious absorption peak, which proved that BBCD catalyzed H_2_O_2_ decomposing into •OH (Supplementary Fig. S9). The degradation of MB in the reaction system containing BBCD and H_2_O_2_ within a prolonged time was used to explore the continuous production of •OH [9]. As presented in Fig. 3a, the gradually decreasing absorption peaks at ∼660 nm were indicative of BBCD interacting with H_2_O_2_ to produce •OH persistently. A significantly enhanced fluorescence intensity was probed at around 430 nm from the oxidation system of TPA with an increasing level of H_2_O_2_, which indicated that the increased concentration of H_2_O_2_ was helpful to •OH generation (Fig. 3b) [9]. BBC had the same ability to react with H_2_O_2_ to produce •OH as BBCD (Supplementary Fig. S10a). On the contrary, no detectable •OH was observed from the system of BB incubating with H_2_O_2_ (Supplementary Fig. S10b). These phenomena revealed that the Cu^2+^ species in BBCD or BBC drove the generation of •OH from H_2_O_2_.

To investigate the reaction process between BBCD and H_2_O_2_, BBCD was incubated with H_2_O_2_ at 37°C for 12 h. The precipitates obtained from centrifugation were labeled as BBCD_H_. It was interesting to find that BBCD nanosphere was losing morphology during incubation of H_2_O_2_, and new crystals grew in the BBCD_H_ (Supplementary Fig. S11). The elements in BBCD_H_ were explored by EDS elemental mapping and XRF analysis. There was no Cu element in BBCD_H_ (Supplementary Figs S12 and S13). The XRD pattern of BBCD_H_ displayed the diffraction peaks assigned to a mixture of bismuth oxide sulfate, which were the new grown crystals in BBCD_H_ (Supplementary Fig. S14). BBCD_H_ was further investigated by XPS analysis (Fig. 3c and d). There were no peaks found in the high-resolution XPS spectra of Cu 2p in BBCD_H_, indicating that there was no Cu in BBCD_H_, which was consistent with the element analysis of BBCD_H_. The peaks of Bi 4f_(Bi__–__S)_ and S 2p_(S_^2^^−^_)_ disappeared in the high-resolution XPS spectra of BBCD_H_. The peaks at 159.3 and 164.6 eV were assigned to Bi 4f_7/2_ and Bi 4f_5/2_ of Bi–O, peaks at 168.4 eV belonged to S 2p of ${\text{SO}}_{4}^{2-}$ [44]. These results suggested that copper-bismuth sulfide nanodots were dissolved by H_2_O_2_ to generate bismuth oxide sulfate and release Cu^2+^, which well explained how the BBCD reacted effectively with H_2_O_2_ to produce •OH.

Then, 5,5′-dithiobis-(2-nitrobenzoic acid) (DTNB) was used as a director to assess the GSH depletion by BBCD. The products from DTNB reacting with GSH displayed a characteristic absorption peak at around 412 nm [45]. As shown in Fig. 3e, BBCD or BBC induced the depletion of GSH directly, while BB could not apparently deplete GSH. And compared with the control group, with the addition of H_2_O_2_, there was a 54% decrease of GSH in the group of BBCD, a 46% reduction of GSH in the group of BBC and only a 9.9% loss of GSH in the group of BB (Fig. 3f). A significant elimination of GSH by BBCD or BBC was realized with the assistance of H_2_O_2_.

The interaction between BBCD and GSH was investigated by XPS analysis. The products of BBCD reacting with GSH were named as BBCD_G_. The binding energies of Cu 2p in BBCD_G_ shifted to 931.8 eV/951.7 eV, which were 0.5 eV lower than the Cu 2p in BBCD (Fig. 3g). When compared with BBCD (Fig. 3h), the binding energies of Bi 4f_Bi__–__S_ in BBCD_G_ moved to the lower at 157.7 eV/163 eV (Δ = −0.5 eV) and the binding energies of S^2^^−^ shifted to the higher at 161.4 eV/162.5 eV (Δ = 0.2 eV). Binding energy was the reflection of electron density, and lower binding energy originated from more outer electrons [46]. The binding energy shifts of Cu 2p and Bi 4f in BBCD_G_ were ascribed to Cu^2+^ and Bi^3+^ coordinating with thiols in GSH [47]. It was concluded that the GSH depletion ability of BBCD was derived from a strong affinity between copper-bismuth sulfide and GSH. As proved in the experiment of BBCD interacting with H_2_O_2_, H_2_O_2_ dismissed the copper-bismuth sulfide nanodots in BBCD to generate bismuth oxide sulfate and release Cu^2+^. The released Cu^2+^ interacted with GSH to generate Cu^+^ and GSSG immediately, and the circular reaction between H_2_O_2_–Cu^2+^–GSH–Cu^+^–H_2_O_2_ initiated the effective depletion of GSH. And the coordination between Bi^3+^ and GSH was in favor of GSH depletion [10]. Since there were copper-bismuth sulfide nanodots in BBCD and BBC, BBCD and BBC exhibited a high efficiency of GSH depletion with the existence of H_2_O_2_. BB had a weak ability to deplete GSH with the assistance of H_2_O_2_, which could be ascribed to the disassembly of BB by H_2_O_2_.

### Amplified oxidative stress by BBCD for enhanced radio-CDT *in vitro*

The physical and chemical properties of BBCD had been evaluated in detail. BBCD showed excellent reactivity with H_2_O_2_ or GSH in solution. The performance of BBCD *in vitro* was explored to assess the therapeutic outcome of combination therapy for 4T1 cancer cells.

**Figure 4.**
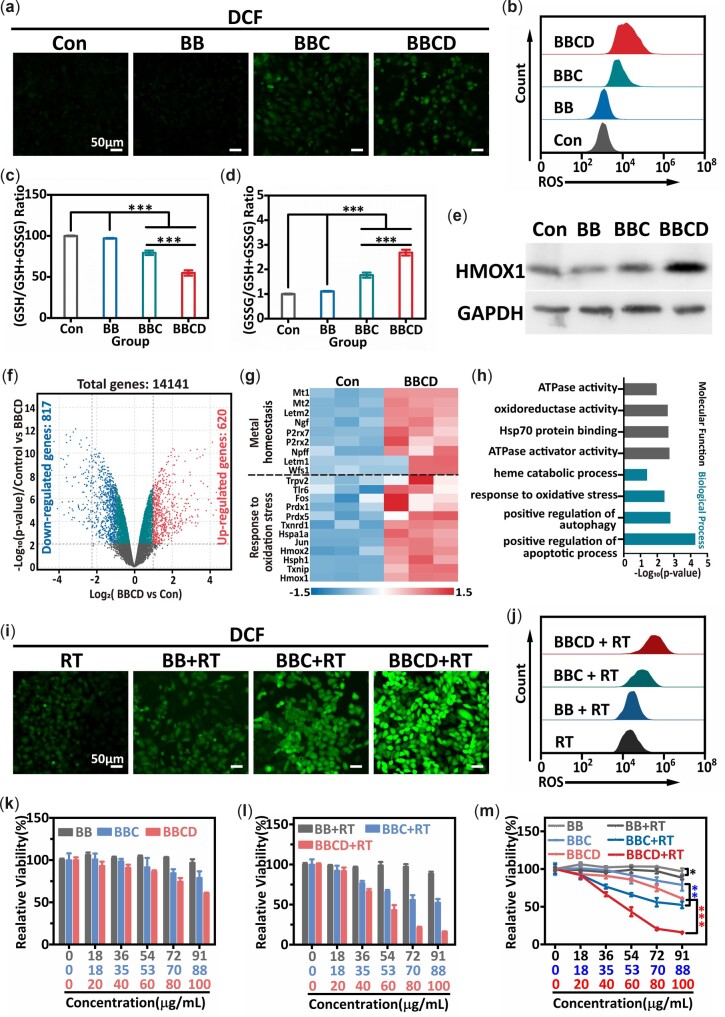
(**a**, **b**) Representative fluorescence images and flow cytometry measurement of ROS in 4T1 cells. The untreated cells as control, [BB] = 91 µg/ml, [BBC] = 88 µg/ml, [BBCD] = 100 µg/ml. (**c**, **d**) Relative GSH and GSSG content in 4T1 cells. The untreated cells as control, [BB] = 91 µg/ml, [BBC] = 88 µg/ml, [BBCD] = 100 µg/ml. (**e**) Western blotting analysis for the expression of HMOX1 in 4T1 cells. The untreated cells as control, [BB] = 91 µg/ml, [BBC] = 88 µg/ml, [BBCD] = 100 µg/ml. RNA-seq for 4T1 cells (control) and BBCD treated 4T1 cells (BBCD). [BBCD] = 100 µg/ml. Differential expression of genes between the control and BBCD groups (**f**). Heatmap for the differential genes related to metal homeostasis and response to oxidative stress in treated 4T1 cells (**g**). GO enrichment analysis for the BBCD treated 4T1 cells (**h**). (**i**, **j**) Representative fluorescence images and flow cytometry detection of ROS in 4T1 cells. [BB] = 91 µg/ml, [BBC] = 88 µg/ml, [BBCD] = 100 µg/ml, X-ray irradiation (RT): 6 Gy. (**k**) The cytotoxicity of BB, BBC or BBCD to 4T1 cells. (**l**) The cancer killing induced by radio-chemodynamic combination therapy. RT (6 Gy) treatment alone as the control group. (**m**) Enhanced therapeutic efficacy *in vitro* (mean ± SD, non-significance was expressed as n.s., and *P <* *0.05, **P <* *0.01, ***P <* *0.001, *n* = 3).

ROS generation and GSH depletion *in vitro* were tested to discuss the interaction between 4T1 cells and BBCD. Intracellular ROS was assessed by the probe of 2′,7′-dichlorodihydrofluorescein diacetate (DCFH-DA), which could be oxidized into DCF with green fluorescence and a stronger fluorescence implying more ROS. Figure 4a shows the ROS levels in treated 4T1 cells. Compared with the BB or untreated group, BBCD or BBC treatment led to a higher level of ROS. BBCD or BBC reacted with intracellular GSH and H_2_O_2_, leading to GSH elimination and •OH production. Furthermore, it was found that DATS treatment could effectively elevate the ROS level in 4T1 cells (Supplementary Fig. S15). This was another important factor for BBCD owning the stronger ability to improve the intracellular ROS level compared with BBC or BB. The ROS concentration in treated 4T1 cells was reconfirmed by measurement of flow cytometry (Fig. 4b), and the variability trends of ROS level were consistent with the result of staining.

Meanwhile, the levels of GSH and GSSG in treated 4T1 cells were determined by the GSH and GSSG assay kit. As demonstrated above, BBCD and BBC exhibited a similar capacity for the elimination of GSH in solution because of the same component of copper-bismuth sulfide. However, BBCD treatment led to more GSH being consumed in 4T1 cells than BBC treatment (Fig. 4c). It was owed to the loaded DATS in BBCD, which could induce the enhancement of ROS in 4T1 cells. And the excess generation of ROS led to more GSH being eliminated [48]. Compared with the BBC treated group, a higher relative GSSG content in 4T1 cells treated by BBCD further confirmed this explanation (Fig. 4d).

Intracellular ROS and GSH played critical roles in redox homeostasis, changes in ROS or GSH levels resulted in redox imbalance and increased oxidative stress [49]. The up-regulated expression of HMOX1 was regarded as a marker of amplified oxidative stress induced by a break in redox balance [50–53]. The western blotting (WB) assay was performed to identify the level of oxidative stress in treated 4T1 cells (Fig. 4e). Compared with the BB or control (untreated) group, the expression of HMOX1 in 4T1 cells treated by BBC or BBCD was apparently up-regulated. Further, HMOX1 in the BBCD treated group was more significantly up-regulated than it was in the BBC group, which was due to the more powerful regulation of ROS and GSH by BBCD in 4T1 cells. The increased oxidative stress achieved by BBCD was reconfirmed by mRNA-sequencing for the 4T1 cells treated by BBCD. In detail, a total of 14141 genes were detected, with 620 up-regulated genes and 817 down-regulated genes (Fig 4f). As shown in Fig. 4g, a large number of genes related to metal homeostasis and oxidative stress response were observed by GO analysis of the differential genes with log2 FC > 1.5, which demonstrated that the BBCD successfully entered the cell and activated the signaling pathway of metal ion metabolism. The differentially expressed genes in the BBCD treated 4T1 cells were mostly associated with oxidative stress-related pathways like autophagy, heme catabolic process, and oxidoreductase activity (Fig. 4h). Taken together, BBCD treatment could increase the oxidative stress of 4T1 cells.

The high level of antioxidative substances in cancer cells was a significant factor for the attenuation of ROS. The decreased GSH and increased ROS for elevated oxidative stress in tumor cells were beneficial for the ROS based therapy [54, 55]. Then, BBCD initiating the elevation of oxidative stress for enhanced radio-CDT was explored *in vitro*. 4T1 cells were pretreated with BB, BBC or BBCD, and then exposed to X-ray irradiation. The intracellular ROS level in treated groups (BB + RT, BBC + RT and BBCD + RT) were detected (Fig. 4i and j). Relative to the group of RT alone, the ROS in the group of BB + RT, BBC + RT or BBCD + RT were apparently elevated, and the maximum level of ROS in the BBCD + RT group was observed. This enhancement of ROS could be attributed to the radiosensitization induced by increased oxidative stress and the high Z element of Bi. GSH was used to investigate the effects of antioxidative substances on ROS production. 4T1 cells were pretreated with GSH + BBCD, then exposed to X-ray irradiation. As displayed in Supplementary Fig. S16, the ROS level in all groups induced by RT was significantly attenuated with the pretreatment of GSH. It implied that increased oxidative stress was conducive to the generation of ROS.

Next, the cell counting kit-8 (CCK-8) assay was used for quantitative analysis of the treatment outcome *in vitro*. The cytotoxicity of BBCD, BBC or BB to 4T1 tumor cells were explored. After 36 h incubation with BBCD (100 µg/ml) or BBC (88 µg/ml), ∼40% and 22% 4T1 cells were killed by BBCD and BBC, respectively, and BB (91 µg/ml) showed no obvious cytotoxicity to 4T1 cells (Fig. 4k). Because the copper-bismuth sulfide nanodots could effectively react with intracellular H_2_O_2_ and GSH to produce •OH and eliminate GSH, BBCD or BBC induced 4T1 cells to be killed in the way of CDT. Due to the loading of DATS efficiently increasing the level of ROS in 4T1 cells, BBCD initiated an enhanced CDT to 4T1 cells [56]. 4T1 cells were incubated with BBCD (100 µg/ml), BBC (88 µg/ml) or BB (91 µg/ml) for 12 h, then exposed to X-ray irradiation and followed by another 24 h incubation for the CCK-8 assay. Compared with the control group (RT alone), 85% 4T1 cells were killed in the group of BBCD (100 µg/ml) + RT, 48% and 12% 4T1 cells were killed in the groups of BBC (88 µg/ml) + RT and BB (91 µg/ml) + RT (Fig. 4l). As displayed in Fig. 4m, BB (91 µg/ml) + RT treatment led to an 8% decrease in cell viability compared with BB (91 µg/ml) treatment alone. There was a 26% reduction in cell viability induced by BBC (88 µg/ml) + RT than by BBC (88 µg/ml), and the cell viability of BBCD (100 µg/ml) + RT group was 45% lower than the BBCD (100 µg/ml) group. The 8% decrease in cell viability between the groups of BB and BB + RT was ascribed to the high Z element of Bi for radiosensitization. The elevated 26% and 45% death of 4T1 cells were assigned to the enhancement of combination therapy induced by increased oxidative stress and high Z element of Bi (Supplementary Fig. S17). Compared with the 8% enhancement in cell killing induced by the high Z element of Bi, BBCD for radio-chemodynamic treatment of 4T1 cells induced a 45% elevation in cell killing to indicate that increased oxidative stress was the main factor for augmented combination therapy.

**Figure 5.**
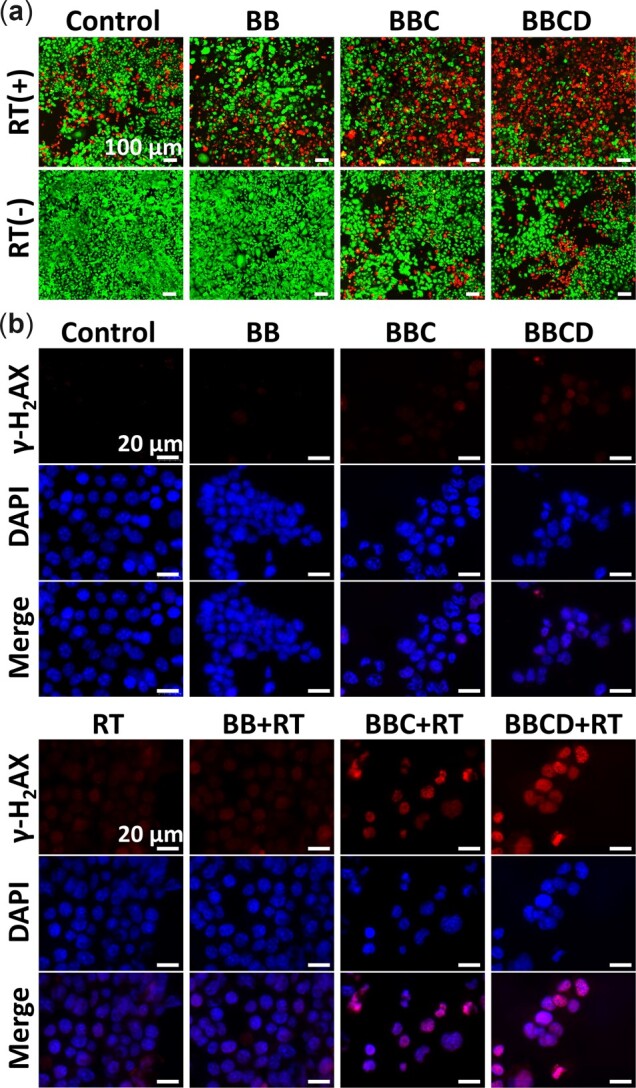
(**a**) Typical images of Calcein-AM and PI staining. No treatment as the control group, [BB] = 91 µg/ml, [BBC] = 88 µg/ml and [BBCD] = 100 µg/ml for 12 h pretreatment, then exposed to X-ray irradiation (0 or 6 Gy) followed by another 24 h incubation. (**b**) Representative images of γ-H_2_AX immunofluorescence for DNA double-strand damage. No treatment as the control group, [BB] = 91 µg/ml, [BBC] = 88 µg/ml, [BBCD] = 100 µg/ml for 12 h pretreatment, then exposed to X-ray irradiation (0 or 2 Gy) followed by another 24 h incubation.

The killing efficiency of 4T1 cells by different treatments were reconfirmed by the live/dead cell staining assay. After different treatments, 4T1 cells were co-stained by calcein-AM and propidium iodide. BBCD combined with RT to kill the most 4T1 cells (Fig. 5a). The damage of DNA double-strand induced by treatments were explored through the staining of γ-H_2_AX immunofluorescence. A brightest red (DNA double-strand breaking) in the 4T1 cells of BBCD + RT group was detected, which implied that BBCD could effectively augment the damage of radiation to DNA (Fig. 5b). Based on the above investigations at the cellular level, an enhanced outcome of radio-chemodynamic combination therapy *in vitro* was achieved by BBCD through the effective regulation of oxidative stress.

### Enhanced radio-CDT induced by BBCD *in vivo*

**Figure 6.**
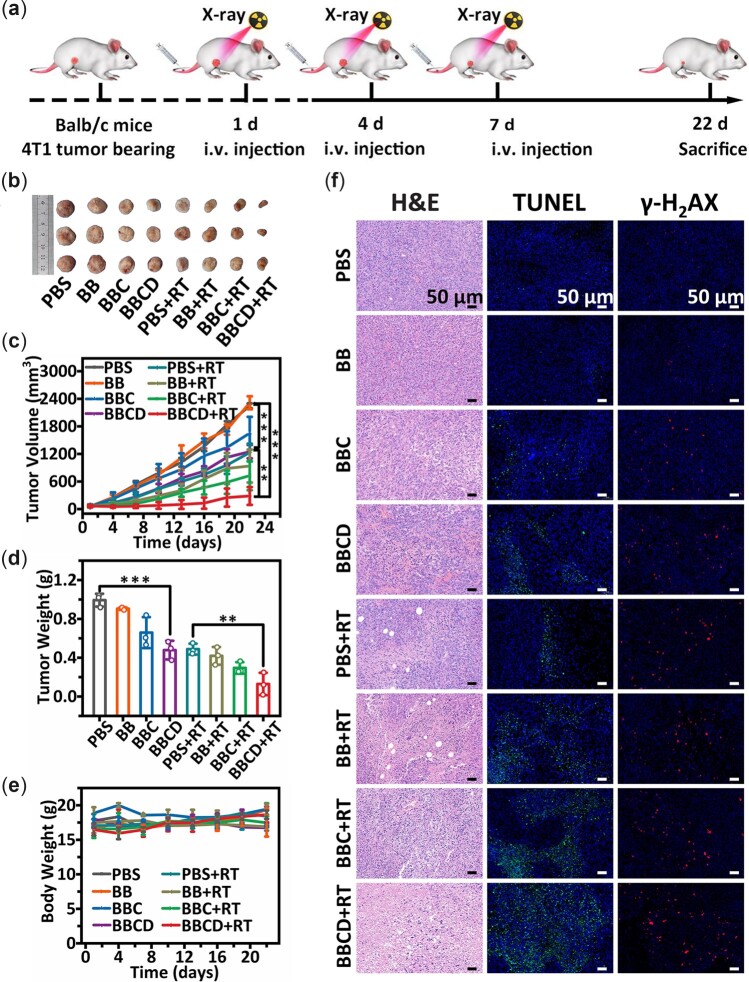
(**a**) Procedure of radio-chemodynamic combination therapy for the 4T1 tumor-bearing balb/c mice. (**b**) A photograph of the dissected tumors from treated mice. (**c**) The volume of tumors during the period of therapy. (**d**) The weight of dissected tumors from mice. (**e**) The body weight of tumor-bearing mice during therapy. (**f**) H&E, TUNEL and γ-H_2_AX immunofluorescence staining results of tumor sections after 21 days of treatment (mean ± SD, non-significance was expressed as n.s., and *P <* *0.05, **P <* *0.01, ***P <* *0.001, *n* = 3).

Based on the experiments *in vitro*, we explored the application of BBCD for radio-chemodynamic combination therapy *in vivo* through the model of subcutaneous 4T1 tumor-bearing Balb/c mice (female, 6 weeks). The mice were randomly divided into eight groups: (i) PBS; (ii) BB; 3 BBC; (iv) BBCD; (v) PBS + RT; (vi) BB + RT; (vii) BBC + RT; (viii) BBCD + RT. All the mice received intravenous injection first, and 18 h later, the mice were exposed to X-ray irradiation. The same treatments were performed on Day 1, 4 and 7 (Fig. 6a).

After 21 days of treatment, the tumor sizes in the group of BBCD + RT were apparently smaller than others (Fig. 6b). BBCD effectively suppressed the growth of tumor, and the BBCD + RT treatment showed the strongest ability to inhibit the growth of tumor *in vivo*. Compared with the treatment of BB or RT, the BB + RT treatment induced a 56% and a 24% decrease in tumor volume, respectively. However, the BBCD + RT treatment induced a 77% and a 74% decrease in tumor volume compared with the treatment of BBCD or RT (Fig. 6c). Furthermore, the tumors in the BBCD + RT treatment group were the lightest among all the groups (Fig. 6d). During the 21 days of treatment, the body weight of all groups showed no significant abnormity (Fig. 6e).

The histological assessment of therapeutic efficacy was performed by H&E staining, TUNEL staining and γ-H_2_AX immunofluorescence staining (Fig. 6f). In comparison to all other groups, the visible contraction of cell nucleus, increased apoptosis and DNA double-strand breaking were observed in the BBCD + RT group. These results demonstrated that BBCD enhanced radio-CDT for the 4T1 tumor *in vivo* by the increased oxidative stress and high Z element.

### Cell viability of BMSC and HUVECs

The acute toxicity of BBCD nanospheres to normal cells was evaluated by the interaction of BBCD with BMSC or HUVECs *in vitro*. The cell viability of BMSC or HUVECs did not change obviously after 24 h of co-incubation with BBCD, indicating that BBCD nanospheres had no cytotoxicity to normal cells (Supplementary Fig. S18).

### Histological observation and blood biochemical analysis

**Figure 7.**
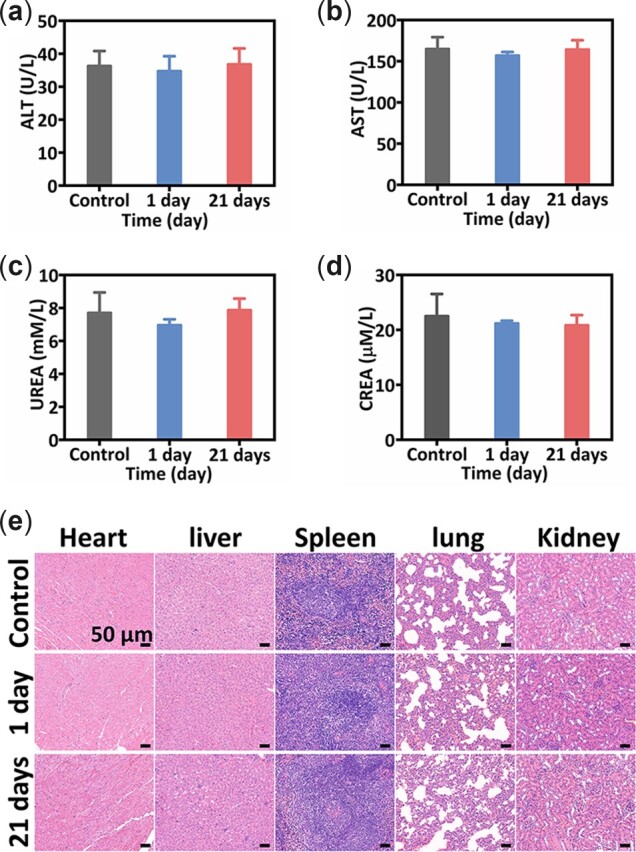
Biochemical analysis of fresh blood from healthy mice (control) and the mice (*n* = 3) treated with BBCD for 1 and 21 days, (**a**) ALT, (**b**) AST, (**c**) CREA, (**d**) UREA. (**e**) H&E staining of major organs (heart, liver, spleen, lung and kidney) from the mice with different treatments.

Then, the long-term toxicity of BBCD nanospheres to normal tissues *in vivo* was investigated through the histological analysis of the main organ as well as biochemical analysis of the blood from the BBCD injected healthy mice. After 1 day or 21 days of injection, fresh blood from the injected mice was collected for the biochemical analysis of alanine aminotransferase (ALT), aspartate aminotransferase (AST), UREA and creatinine (CREA). Compared with healthy Balb/c mice (control), the ALT and AST data of liver function for injected mice (1 and 21 days) showed no abnormality, as did the UREA and CREA indexes of kidney function (Fig. 7a–d). Compared with the healthy mice (control), no obvious damage to major organs was observed from the results of H&E staining (Fig. 7e). As demonstrated by the investigations *in vitro* and *in vivo*, BBCD nanospheres were safe for the application of tumor therapy.

## Conclusion

In summary, BBCD bioactive nanospheres were successfully constructed through an *in situ* assembly approach by BSA, copper-bismuth sulfide nanodots and DATS. It was demonstrated that those *in situ* grown copper-bismuth sulfide nanodots in the BBCD nanosphere could interact with the overexpressed H_2_O_2_ and GSH in 4T1 cells to achieve CDT via •OH generation and GSH elimination. At the same time, the DTAS from BBCD nanospheres could further induce the elevation of ROS in 4T1 cells. The efficient modulation of ROS and GSH by BBCD resulted in the breaking of antioxidative system for a significant up-regulation of intracellular oxidative stress, which enhanced the sensitivity of 4T1 tumor cells to radio-CDT *in vitro* and *in vivo*. In a subcutaneous 4T1 tumor model, the enhanced radio-CDT was confirmed by the increased contraction of cell nucleus, cell apoptosis and DNA double-strand breaking in the smallest tumor tissue obtained by the treatment of BBCD combined with RT. Furthermore, the results from the evaluation of cytotoxicity to normal cells, histological evaluation of major organs and blood biochemical analysis of the BBCD injected mice indicated that BBCD nanospheres were nontoxic to normal cells and tissues. Therefore, such well-designed BBCD nanospheres achieved the enhancement of radio-CDT by regulation of intracellular oxidative stress, which provided a novel prospective strategy for enhanced solid tumor complex therapy.

## Supplementary data

Supplementary data are available at *REGBIO* online.

## Supplementary Material

rbac045_Supplementary_Data

## References

[1] Yang B , Chen Y, Shi J. Reactive oxygen species (ROS)-based nanomedicine. Chem Rev 2019;119:4881–985. 30973011 10.1021/acs.chemrev.8b00626

[2] Su L , Zhang J, Gomez H, Murugan R, Hong X, Xu D, Jiang F, Peng Z. Reactive oxygen species-induced lipid peroxidation in apoptosis, autophagy, and ferroptosis. Oxid Med Cell Longev 2019;2019:5080843. 31737171 10.1155/2019/5080843 PMC6815535

[3] Srinivas US , Tan BWQ, Vellayappan BA, Jeyasekharan AD. ROS and the DNA damage response in cancer. Redox Biol 2019;25:101084. 30612957 10.1016/j.redox.2018.101084 PMC6859528

[4] Costantini D , Verhulst S. Does high antioxidant capacity indicate low oxidative stress? Funct Ecol 2009;23:506–9.

[5] Asaduzzaman Khan M , Tania M, Zhang D, Chen H. Antioxidant enzymes and cancer. Chin J Cancer Res 2010;22:87–92.

[6] He L , He T, Farrar S, Ji L, Liu T, Ma X. Antioxidants maintain cellular redox homeostasis by elimination of reactive oxygen species. Cell Physiol Biochem 2017;44:532–53. 29145191 10.1159/000485089

[7] Sies H. Glutathione and its role in cellular functions. Free Radical Bio Med 1999;27:916–21. 10569624 10.1016/s0891-5849(99)00177-x

[8] Wu G , Fang YZ, Yang S, Lupton JR, Turner ND. Glutathione metabolism and its implications for health. J Nutr 2004;134:489–92. 14988435 10.1093/jn/134.3.489

[9] Ma B , Wang S, Liu F, Zhang S, Duan J, Li Z, Kong Y, Sang Y, Liu H, Bu W, Li L. Self-assembled copper-amino acid nanoparticles for in situ glutathione “and” H_2_O_2_ sequentially triggered chemodynamic therapy. J Am Chem Soc 2019;141:849–57. 30541274 10.1021/jacs.8b08714

[10] Liu J , Zhang J, Song K, Du J, Wang X, Liu J, Li B, Ouyang R, Miao Y, Sun Y, Li Y. Tumor microenvironment modulation platform based on composite biodegradable bismuth–manganese radiosensitizer for inhibiting radioresistant hypoxic tumors. Small 2021;17:2101015. 10.1002/smll.202101015 34263544

[11] Wang S , Wang Z, Yu G, Zhou Z, Jacobson O, Liu Y, Ma Y, Zhang F, Chen Z, Chen X. Tumor-specific drug release and reactive oxygen species generation for cancer chemo/chemodynamic combination therapy. Adv Sci (Weinh) 2019;6:1801986. 30886808 10.1002/advs.201801986 PMC6402284

[12] Xiao S , Lu Y, Feng M, Dong M, Cao Z, Zhang X, Chen Y, Liu J. Multifunctional FeS_2_ theranostic nanoparticles for photothermal-enhanced chemodynamic/photodynamic cancer therapy and photoacoustic imaging. Chem Eng J 2020;396:125294.

[13] Wang Y , Wang J, Hao H, Cai M, Wang S, Ma J, Li Y, Mao C, Zhang S. In vitro and in vivo mechanism of bone tumor inhibition by selenium-doped bone mineral nanoparticles. ACS Nano 2016;10:9927–37. 27797178 10.1021/acsnano.6b03835 PMC5198771

[14] Liu C , Sun S, Feng Q, Wu G, Wu Y, Kong N, Yu Z, Yao J, Zhang X, Chen W, Tang Z, Xiao Y, Huang X, Lv A, Yao C, Cheng H, Wu A, Xie T, Tao W. Arsenene nanodots with selective killing effects and their low-dose combination with ß-elemene for cancer therapy. Adv Mater 2021;33:2102054. 10.1002/adma.202102054 34309925

[15] Liu C , Shin J, Son S, Choe Y, Farokhzad N, Tang Z, Xiao Y, Kong N, Xie T, Kim JS, Tao W. Pnictogens in medicinal chemistry: evolution from erstwhile drugs to emerging layered photonic nanomedicine. Chem Soc Rev 2021;50:2260–79. 33367452 10.1039/d0cs01175d

[16] Ji X , Ge L, Liu C, Tang Z, Xiao Y, Chen W, Lei Z, Gao W, Blake S, De D, Shi B, Zeng X, Kong N, Zhang X, Tao W. Capturing functional two-dimensional nanosheets from sandwich-structure vermiculite for cancer theranostics. Nat Commun 2021;12:1124. 33602928 10.1038/s41467-021-21436-5 PMC7892577

[17] Tang Z , Zhao P, Wang H, Liu Y, Bu W. Biomedicine meets Fenton chemistry. Chem Rev 2021;121:1981–2019. 33492935 10.1021/acs.chemrev.0c00977

[18] Zhao P , Jiang Y, Tang Z, Li Y, Sun B, Wu Y, Wu J, Liu Y, Bu W. Constructing electron levers in perovskite nanocrystals to regulate the local electron density for intensive chemodynamic therapy. Angew Chem 2021;133:8987–94. 10.1002/anie.202100864 33527642

[19] Koo S , Park OK, Kim J, Han SI, Yoo TY, Lee N, Kim YG, Kim H, Lim C, Bae JS, Yoo J, Kim D, Choi SH, Hyeon T. Enhanced chemodynamic therapy by Cu–Fe peroxide nanoparticles: tumor microenvironment-mediated synergistic Fenton reaction. ACS Nano 2022;16:2535–45. 35080370 10.1021/acsnano.1c09171

[20] Liu C , Cao Y, Cheng Y, Wang D, Xu T, Su L, Zhang X, Dong H. An open source and reduce expenditure ROS generation strategy for chemodynamic/photodynamic synergistic therapy. Nat Commun 2020;11:1735. 32269223 10.1038/s41467-020-15591-4 PMC7142144

[21] Vaupel P. Tumor microenvironmental physiology and its implications for radiation oncology. Semin Radiat Oncol 2004;14:198–206. 15254862 10.1016/j.semradonc.2004.04.008

[22] Li Y , Gong T, Gao H, Chen Y, Li H, Zhao P, Jiang Y, Wang K, Wu Y, Zheng X, Bu W. ZIF-based nanoparticles combine X-ray-induced nitrosative stress with autophagy management for hypoxic prostate cancer therapy. Angew Chem Int Ed Engl 2021;60:15472–81. 33964189 10.1002/anie.202103015

[23] Lv B , Zhang H, Zheng X, Wang H, Ge W, Ren Y, Tan Z, Zhang M, Tang Z, Liu Y, Zhang L, Wu Y, Jiang X, Bu W. Structure-oriented catalytic radiosensitization for cancer radiotherapy. Nano Today 2020;35:100988.

[24] Wang X , Guo Z, Zhang C, Zhu S, Li L, Gu Z, Zhao Y. Ultrasmall BiOI quantum dots with efficient renal clearance for enhanced radiotherapy of cancer. Adv Sci (Weinh) 2020;7:1902561. 32195085 10.1002/advs.201902561 PMC7080545

[25] Li Y , Yun K, Lee H, Goh S, Suh YG, Choi Y. Porous platinum nanoparticles as a high-Z and oxygen generating nanozyme for enhanced radiotherapy in vivo. Biomaterials 2019;197:12–9. 30623793 10.1016/j.biomaterials.2019.01.004

[26] Zhou R , Wang H, Yang Y, Zhang C, Dong X, Du J, Yan L, Zhang G, Gu Z, Zhao Y. Tumor microenvironment-manipulated radiocatalytic sensitizer based on bismuth heteropolytungstate for radiotherapy enhancement. Biomaterials 2019;189:11–22. 30384125 10.1016/j.biomaterials.2018.10.016

[27] Chai R , Yu L, Dong C, Yin Y, Wang S, Chen Y, Zhang Q. Oxygen-evolving photosynthetic cyanobacteria for 2D bismuthene radiosensitizer-enhanced cancer radiotherapy. Bioact Mater 2022;17:276–88. 35386463 10.1016/j.bioactmat.2022.01.014 PMC8965086

[28] Dai X , Ruan J, Guo Y, Sun Z, Liu J, Bao X, Zhang H, Li Q, Ye C, Wang X, Zhao C, Zhou F, Sheng J, Chen D, Zhao P. Enhanced radiotherapy efficacy and induced anti-tumor immunity in HCC by improving hypoxia microenvironment using oxygen microcapsules. Chem Eng J 2021;422:130109.

[29] Trachootham D , Alexandre J, Huang P. Targeting cancer cells by ROS-mediated mechanisms: a radical therapeutic approach? Nat Rev Drug Discov 2009;8:579–91. 19478820 10.1038/nrd2803

[30] Dong Z , Feng L, Chao Y, Hao Y, Chen M, Gong F, Han X, Zhang R, Cheng L, Liu Z. Amplification of tumor oxidative stresses with liposomal Fenton catalyst and glutathione inhibitor for enhanced cancer chemotherapy and radiotherapy. Nano Lett 2019;19:805–15. 30592897 10.1021/acs.nanolett.8b03905

[31] Cheng R , Liu H, Dong X, Zhu S, Zhou R, Wang C, Wang Y, Wang X, Su C, Gu Z. Semiconductor heterojunction-based radiocatalytic platforms for tumors treatment by enhancing radiation response and reducing radioresistance. Chem Eng J 2020;394:124872.

[32] Zhang Q , Chen J, Ma M, Wang H, Chen H. A bioenvironment-responsive versatile nanoplatform enabling rapid clearance and effective tumor homing for oxygen-enhanced radiotherapy. Chem Mater 2018;30:5412–21.

[33] Long B , Qiao Z, Zhang J, Zhang S, Balogun MS, Lu J, Song S, Tong Y. Polypyrrole-encapsulated amorphous Bi_2_S_3_ hollow sphere for long life sodium ion batteries and lithium–sulfur batteries. J Mater Chem A 2019;7:11370–8.

[34] Yu Y , Zhang B, Ge Z, Shang P, Chen Y. Thermoelectric properties of Ag-doped bismuth sulfide polycrystals prepared by mechanical alloying and spark plasma sintering. Mater Chem Phys 2011;131:216–22.

[35] Liu Y , Li M, Zhang Q, Qin P, Wang X, He G, Li L. One-step synthesis of a WO_3_-CuS nanosheet heterojunction with enhanced photocatalytic performance for methylene blue degradation and Cr(VI) reduction. J Chem Technol Biotechnol 2020;95:665–74.

[36] Ren X , Yuan L, Liang Q, Xie R, Geng Z, Sun Y, Wang L, Huang K, Wu T, Feng S. Phase-controlled synthesis of high-Bi-ratio ternary sulfide nanocrystals of Cu_1.57_Bi_4.57_S_8_ and Cu_2.93_Bi_4.89_S_9_. Chempluschem 2018;83:812–8. 31950663 10.1002/cplu.201800271

[37] Liang H , Shuang W, Zhang Y, Chao S, Han H, Wang X, Zhang H, Yang L. Graphene-like multilayered CuS nanosheets assembled into flower-like microspheres and their electrocatalytic oxygen evolution properties. ChemElectroChem 2018;5:494–500.

[38] Weng G , Yang B, Liu C, Du G, Li EY, Lu Y. Asymmetric allyl-activation of organosulfides for high-energy reversible redox flow batteries. Energy Environ Sci 2019;12:2244–52.

[39] Huang N , Xia Q, Zhang Z, Zhao L, Zhang G, Gao J, Tang L. Simultaneous improvements in fire resistance and alarm response of GO paper via one-step 3-mercaptopropyltrimethoxysilane functionalization for efficient fire safety and prevention. Compos Part A-Appl S 2020;131:105797.

[40] Liu Y , Liu H, Wang L, Wang Y, Zhang C, Wang C, Yan Y, Fan J, Xu G, Zhang Q. Amplification of oxidative stress via intracellular ROS production and antioxidant consumption by two natural drug-encapsulated nanoagents for efficient anticancer therapy. Nanoscale Adv 2020;2:3872–81. 10.1039/d0na00301h PMC9419310 36132787

[41] Zheng Z , Chen Q, Dai R, Jia Z, Yang C, Peng X, Zhang R. A continuous stimuli-responsive system for NIR-II fluorescence/photoacoustic imaging guided photothermal/gas synergistic therapy. Nanoscale 2020;12:11562–72. 32432283 10.1039/d0nr02543g

[42] Lin L , Huang T, Song J, Ou X, Wang Z, Deng H, Tian R, Liu Y, Wang J, Liu Y, Yu G, Zhou Z, Wang S, Niu G, Yang H, Chen X. Synthesis of copper peroxide nanodots for H_2_O_2_ self-supplying chemodynamic therapy. J Am Chem Soc 2019;141:9937–45. 31199131 10.1021/jacs.9b03457

[43] Wang T , Zhang H, Liu H, Yuan Q, Ren F, Han Y, Sun Q, Li Z, Gao M. Boosting H_2_O_2_-guided chemodynamic therapy of cancer by enhancing reaction kinetics through versatile biomimetic Fenton nanocatalysts and the second near-infrared light irradiation. Adv Funct Mater 2020;30:1906128.

[44] Yi H , Li K, Tang X, Ning P, Peng J, Wang C, He D. Simultaneous catalytic hydrolysis of low concentration of carbonyl sulfide and carbon disulfide by impregnated microwave activated carbon at low temperatures. Chem Eng J 2013;230:220–6.

[45] Wang Z , Liu B, Sun Q, Dong S, Kuang Y, Dong Y, He F, Gai S, Yang P. Fusiform-like copper(II)-based metal-organic framework through relief hypoxia and GSH-depletion co-enhanced starvation and chemodynamic synergetic cancer therapy. ACS Appl Mater Interfaces 2020;12:17254–67. 32227859 10.1021/acsami.0c01539

[46] Zhang P , Li Y, Zhang Y, Hou R, Zhang X, Xue C, Wang S, Zhu B, Li N, Shao G. Photogenerated electron transfer process in heterojunctions: in situ irradiation XPS. Small Methods 2020;4:2000214.

[47] Zhang Q , Dai Z, Cheng G, Liu Y, Chen R. In-situ room-temperature synthesis of amorphous/crystalline contact Bi_2_S_3_/Bi_2_WO_6_ heterostructures for improved photocatalytic ability. Ceram Int 2017;43:11296–304.

[48] Niu B , Liao K, Zhou Y, Wen T, Quan G, Pan X, Wu C. Application of glutathione depletion in cancer therapy: enhanced ROS-based therapy, ferroptosis, and chemotherapy. Biomaterials 2021;277:121110. 34482088 10.1016/j.biomaterials.2021.121110

[49] Gorrini C , Harris IS, Mak TW. Modulation of oxidative stress as an anticancer strategy. Nat Rev Drug Discov 2013;12:931–47. 24287781 10.1038/nrd4002

[50] Cao K , Du Y, Bao X, Han M, Su R, Pang J, Liu S, Shi Z, Yan F, Feng S. Glutathione-bioimprinted nanoparticles targeting of N6-methyladenosine FTO demethylase as a strategy against leukemic stem cells. Small 2022;18:2106558. 10.1002/smll.202106558 35119204

[51] Sarma SN , Kim YJ, Song M, Ryu JC. Induction of apoptosis in human leukemia cells through the production of reactive oxygen species and activation of HMOX1 and Noxa by benzene, toluene, and o-xylene. Toxicology 2011;280:109–17. 21144877 10.1016/j.tox.2010.11.017

[52] Hufnagel M , Schoch S, Wall J, Strauch BM, Hartwig A. Toxicity and gene expression profiling of copper- and titanium-based nanoparticles using air–liquid interface exposure. Chem Res Toxicol 2020;33:1237–49. 32285662 10.1021/acs.chemrestox.9b00489

[53] Li K , Xu K, He Y, Lu L, Mao Y, Gao P, Liu G, Wu J, Zhang Y, Xiang Y, Luo Z, Cai K. Functionalized tumor-targeting nanosheets exhibiting Fe(ii) overloading and GSH consumption for ferroptosis activation in liver tumor. Small 2021;17:2102046. 10.1002/smll.202102046 34448349

[54] Liu J , Wu M, Pan Y, Duan Y, Dong Z, Chao Y, Liu Z, Liu B. Biodegradable nanoscale coordination polymers for targeted tumor combination therapy with oxidative stress amplification. Adv Funct Mater 2020;30:1908865.

[55] Yong Y , Zhang C, Gu Z, Du J, Guo Z, Dong X, Xie J, Zhang G, Liu X, Zhao Y. Polyoxometalate-based radiosensitization platform for treating hypoxic tumors by attenuating radioresistance and enhancing radiation response. ACS Nano 2017;11:7164–76. 28640996 10.1021/acsnano.7b03037

[56] Yu X , Lu M, Luo Y, Hu Y, Zhang Y, Xu Z, Gong S, Wu Y, Ma X, Yu B, Tian J. A cancer-specific activatable theranostic nanodrug for enhanced therapeutic efficacy via amplification of oxidative stress. Theranostics 2020;10:371–83. 31903126 10.7150/thno.39412 PMC6929611

