# Supplementary material for: Albumin-assembled copper-bismuth bimetallic sulfide bioactive nanosphere as an amplifier of oxidative stress for enhanced radio-chemodynamic combination therapy
Source: Regen Biomater. 2022 Jul 5;9:rbac045. doi: 10.1093/rb/rbac045 (PMC9290530; doi:10.1093/rb/rbac045)
Supplement: rbac045_Supplementary_Data [file rbac045_supplementary_data.docx]

**Albumin-assembled Copper-Bismuth Bimetallic Sulfide Bioactive Nanosphere as an Amplifier of Oxidative Stress for Enhanced Radio-Chemodynamic Combination Therapy**

Weiyong Tao ^a, b, c, 1^, Zhan Tuo ^d, 1^, Feige Wu ^a, b, c^, Ketao Mu ^e^, Cunjing Xu ^a, b, c^, Yuxiao Shi ^a, b, c^, Zeyu Sun ^a, b, c^, Yifan Wang ^a, b, c^, Yan Li ^a, b, c^, Zhenyu Zhong ^a, b, c^, Lei Zhou ^a, b, c^,

Jianglin Wang ^a, b, c^, Jie Liu ^f, *^, Yingying Du ^a, b, c, **^, Shengmin Zhang ^a, b, c, ***^

^a^ Advanced Biomaterials and Tissue Engineering Center, Huazhong University of Science and Technology, Wuhan, 430074, China

^b^ NMPA Research Base of Regulatory Science for Medical Devices & Institute of Regulatory Science for Medical Devices, Huazhong University of Science and Technology, Wuhan 430074, China

^c^ Department of Biomedical Engineering, Huazhong University of Science and Technology, Wuhan 430074, China

^d^ Cancer Center, Union Hospital, Tongji Medical College, Huazhong University of Science and Technology, Wuhan 430022, China

^e^ Department of Radiology, Tongji Hospital, Tongji Medical College, Huazhong University of Science and Technology, Wuhan 430030, China

^f^ School of Biomedical Engineering, Sun Yat-sen University, Guangzhou, Guangdong 510006, China

* Corresponding author: School of Biomedical Engineering, Sun Yat-sen University, Guangzhou, Guangdong 510006, China

** Corresponding author: Advanced Biomaterials and Tissue Engineering Center, Huazhong University of Science and Technology, Wuhan, 430074, China

*** Corresponding author: Advanced Biomaterials and Tissue Engineering Center, Huazhong University of Science and Technology, Wuhan, 430074, China

E-mail addresses: liujie56@mail.sysu.edu.cn (J. Liu), yingyingdu@hust.edu.cn (Y. Du), smzhang@hust.edu.cn (S. Zhang).

^1^ These authors contributed equally to this work.

**Keywords:** Bioactive materials; Nanosphere; Assembly; Bismuth-Copper; Radio-chemodynamic therapy


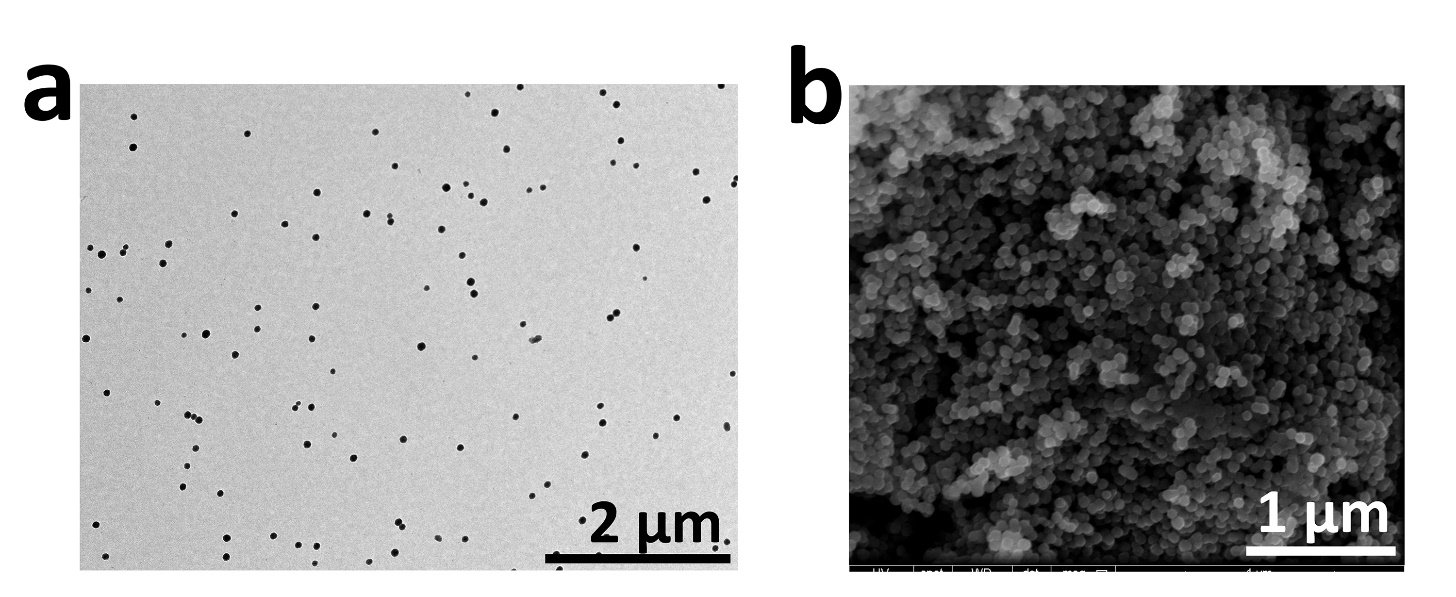


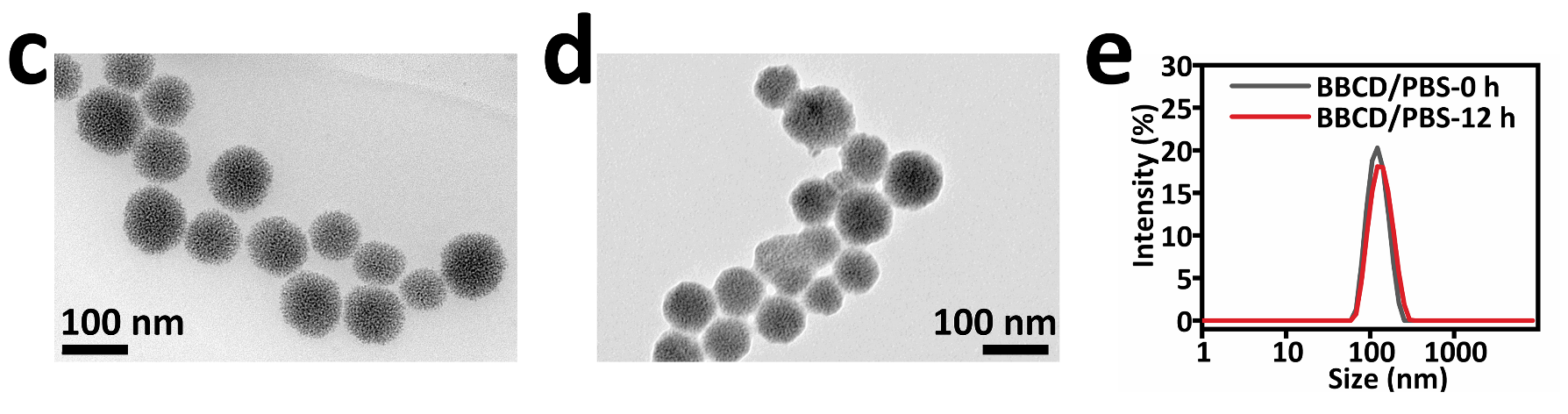


Fig. S1. a) TEM and b) SEM images of BBCD nanospheres. BBCD in PBS for c) 0 h and d) 12 h, e) Hydrodynamic sizes of BBCD in PBS.


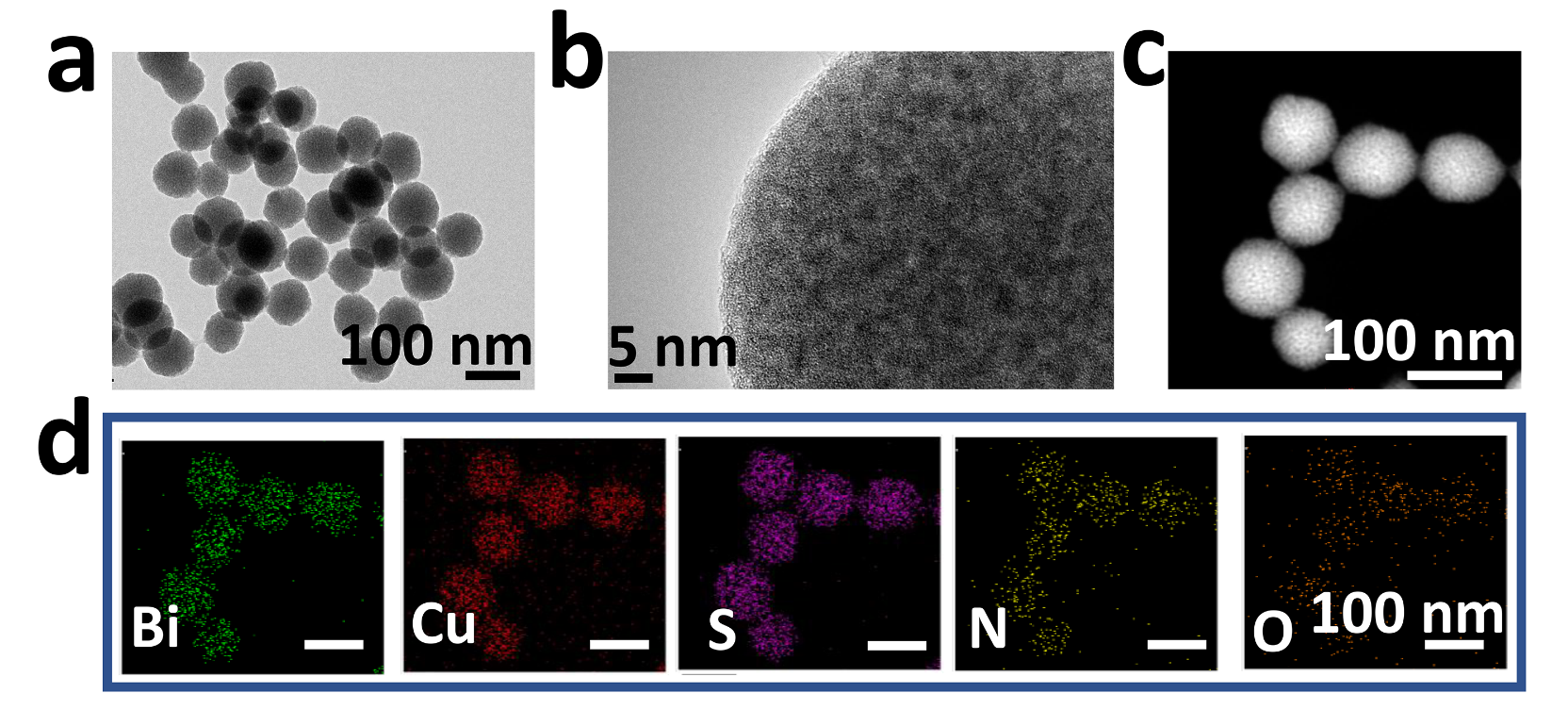


Fig. S2. a) TEM image of BBC nanospheres. b) HRTEM image of BBC nanosphere (no lattice fringes). c) HADDF image of BBC nanospheres.d) EDS elements mappings of BBC nanosphere (BBC: copper-bismuth sulfide (BC) nanodots loaded in BSA nanosphere).


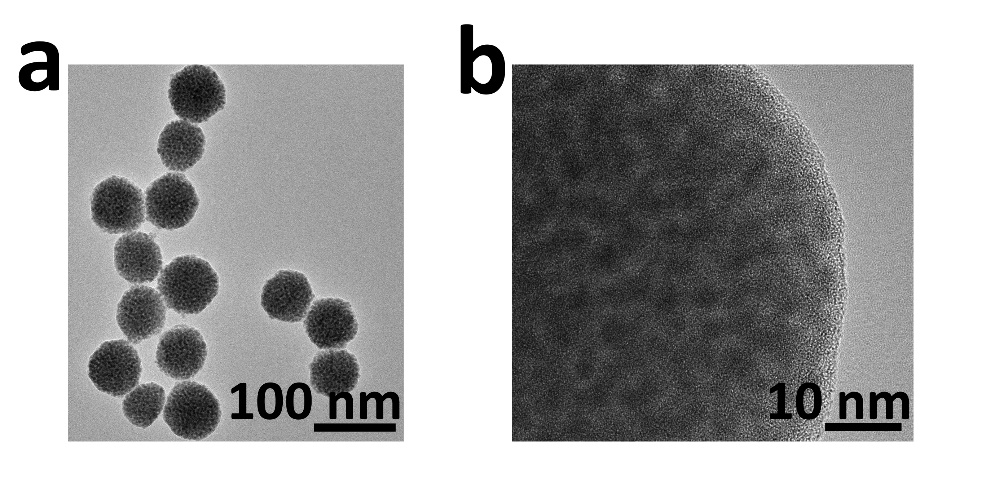


Fig. S3. a) TEM of BB nanospheres. b) HRTEM image of BB nanosphere. (BB: bismuth sulfide (BC) nanodots loaded in BSA nanosphere).


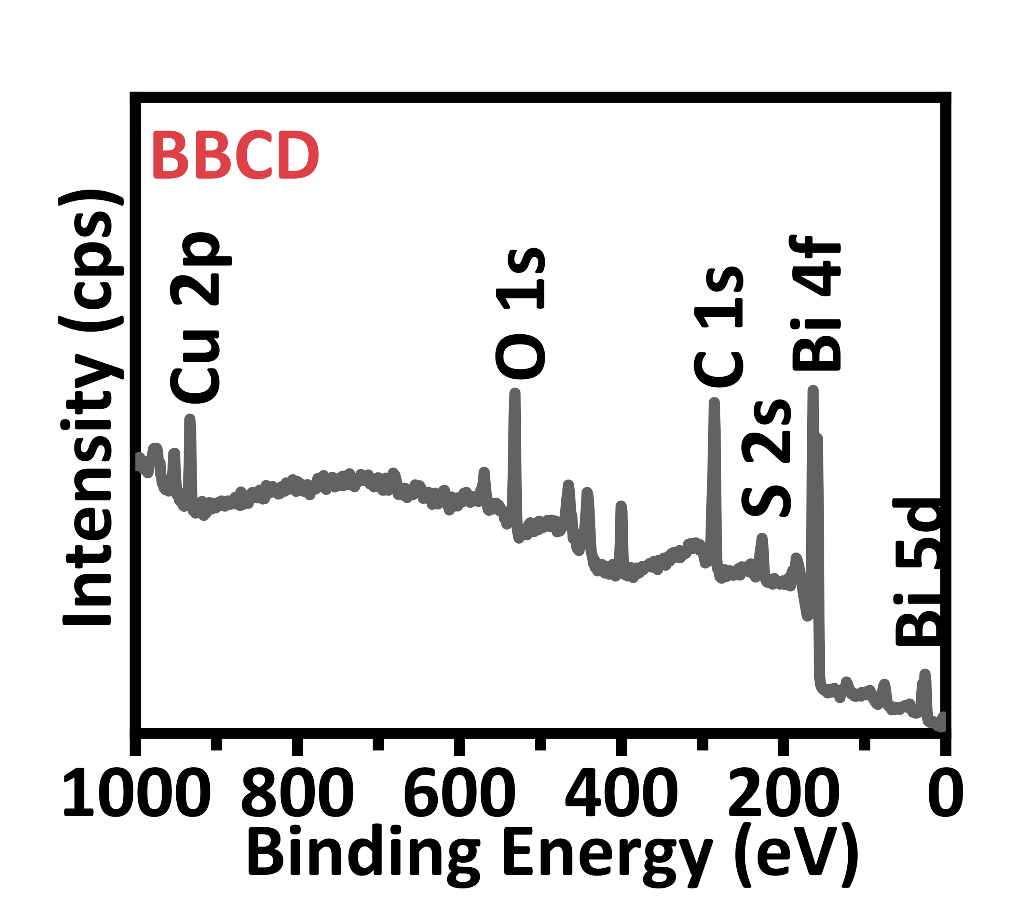


Fig. S4. Wide spectrum of XPS analysis for BBCD nanospheres (There existed Bi, Cu, S, O in BBCD nanosphere).


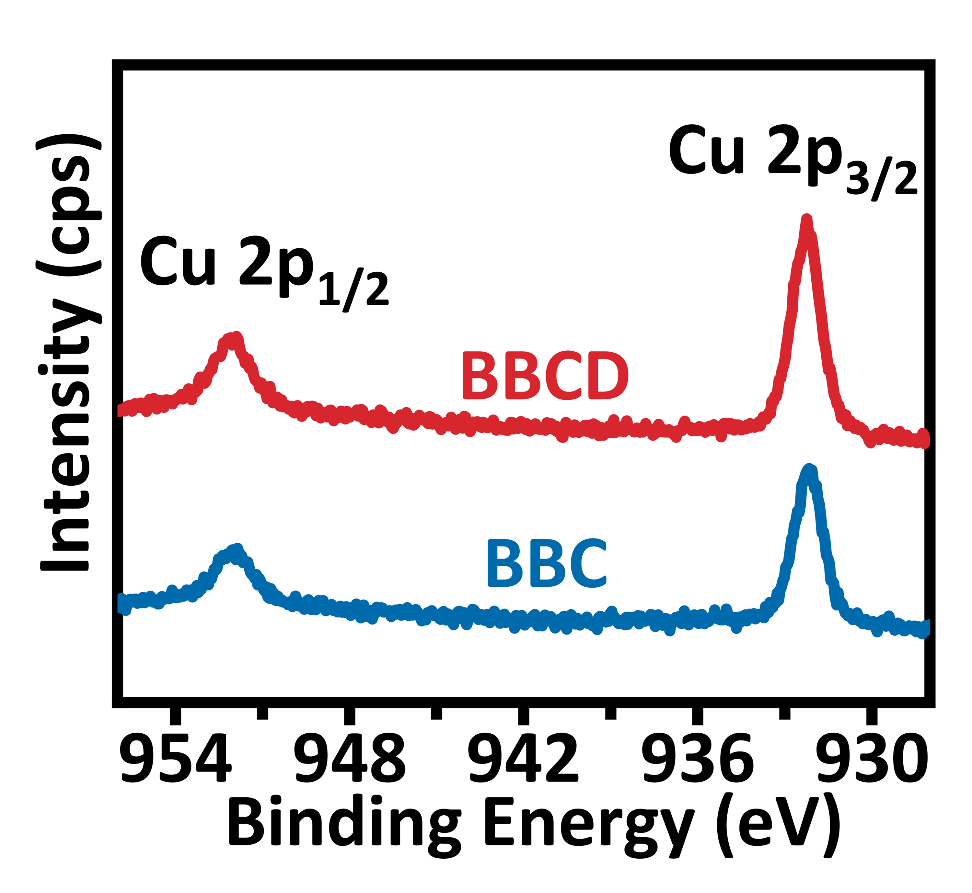


Fig. S5. High-resolution XPS spectra of Cu 2p in BBCD and BBC nanospheres. Cu 2p in BBCD located at 932.3 eV and 952.2 eV, Cu 2p in BBC located at 932. 1 eV and 952 eV.


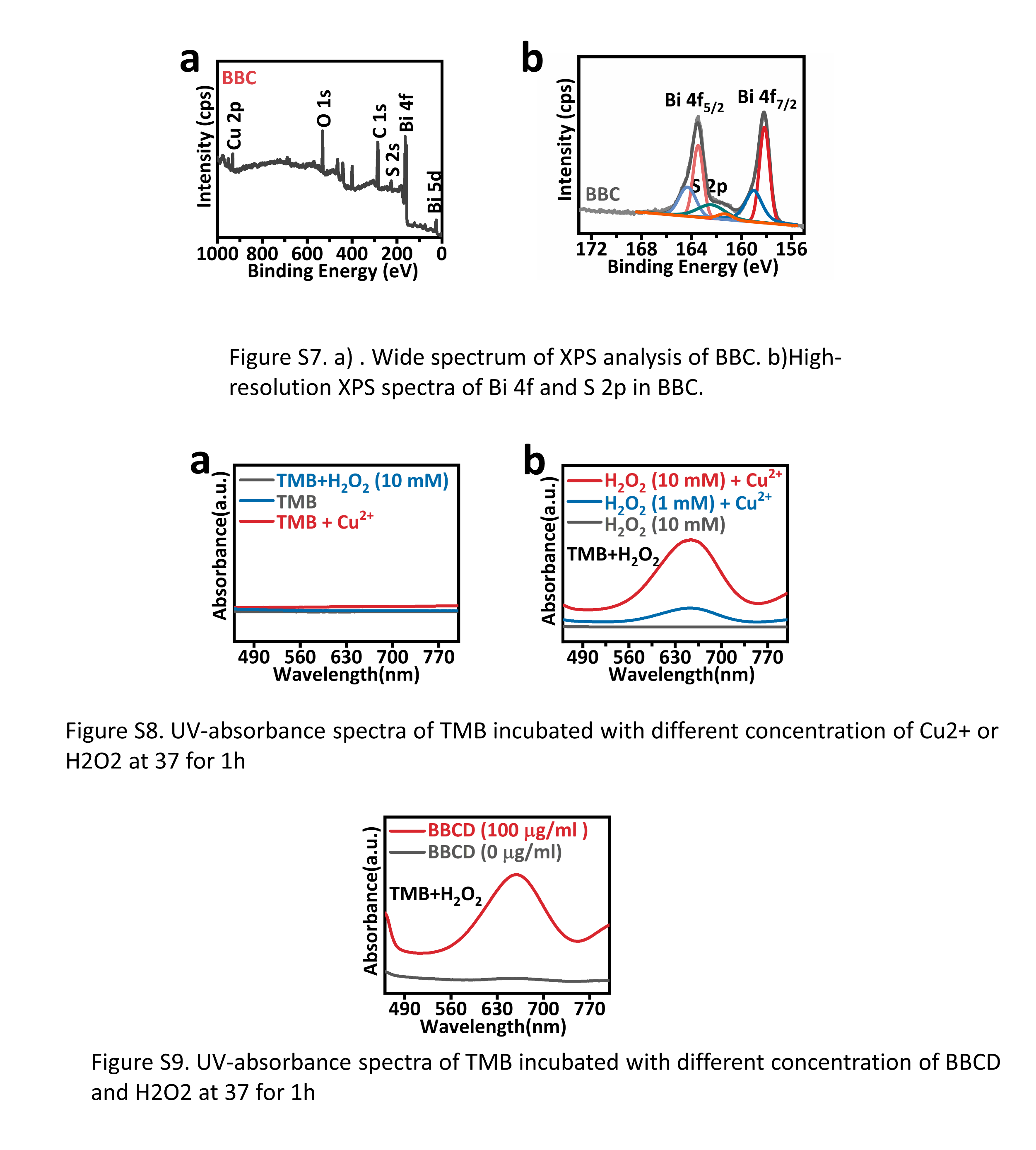


Fig. S6. a) Wide spectrum of XPS analysis for BBC nanospheres. b)High-resolution XPS spectra of Bi 4f and S 2p in BBC. Peaks at 158.1 eV and 163.4 eV were attributed to Bi 4f7/2 and Bi 4f5/2 of Bi-S in BBC, 158.9 eV and 164.2 eV were assigned to the Bi-S oxidation at the surface of nanodots, 161.3 eV, 162.4 eV were ascribed to binding energies of S2-.


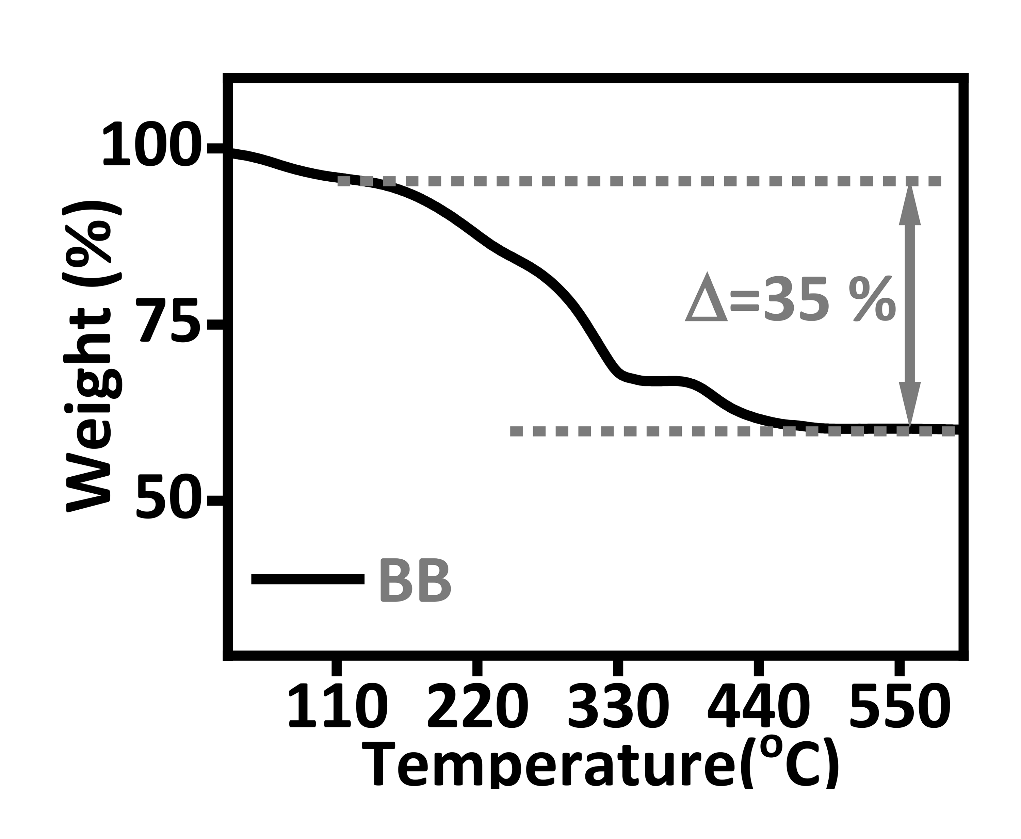


Fig. S7. TG analysis of BB. Operation with an increasing rate of 20 °C/min from room temperature to 650 °C under air atmosphere.


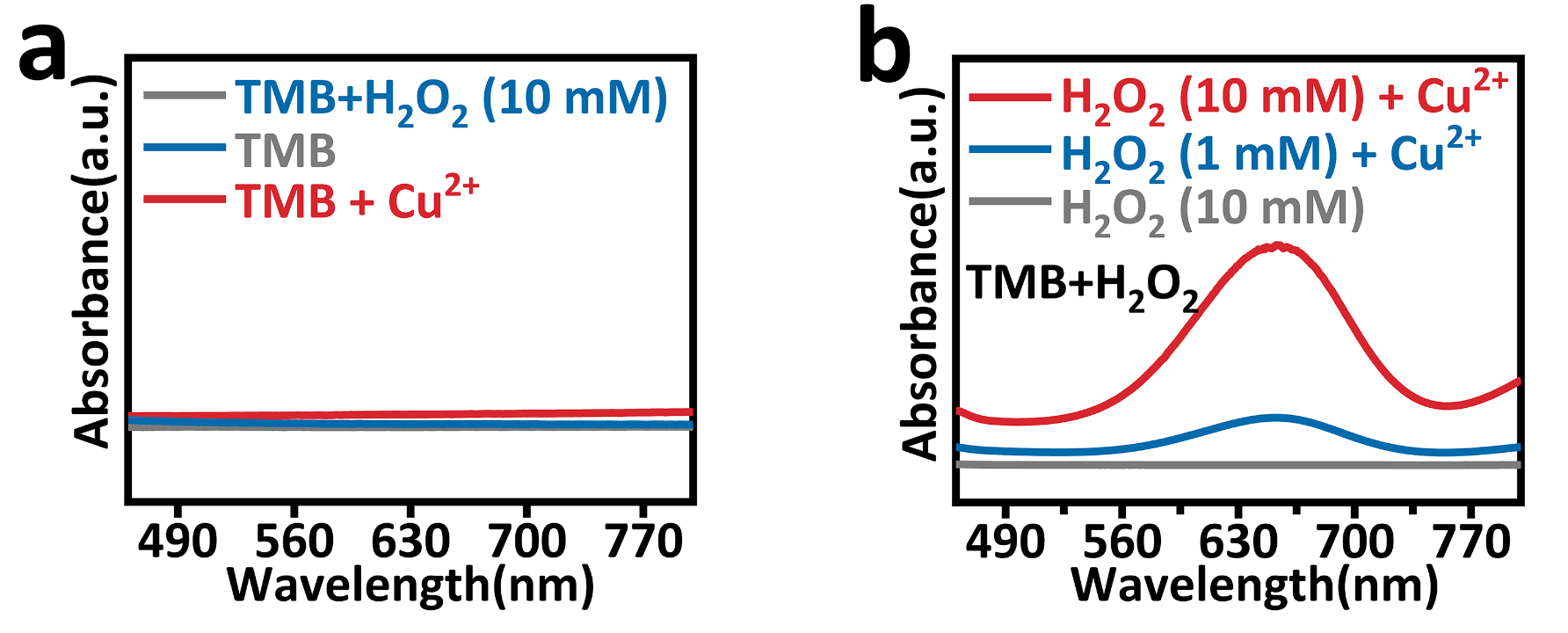


Fig. S8. UV-absorbance spectra of TMB for detection of •OH production. TMB incubated with different concentration of Cu^2+^ or H_2_O_2_ at 37 °C for 1 h.





Fig. S9. UV-absorbance spectra of TMB for detection of •OH production. TMB incubated with different concentration of BBCD and H_2_O_2_ = [10 mM] at 37 °C for 1 h to detect the production of •OH.


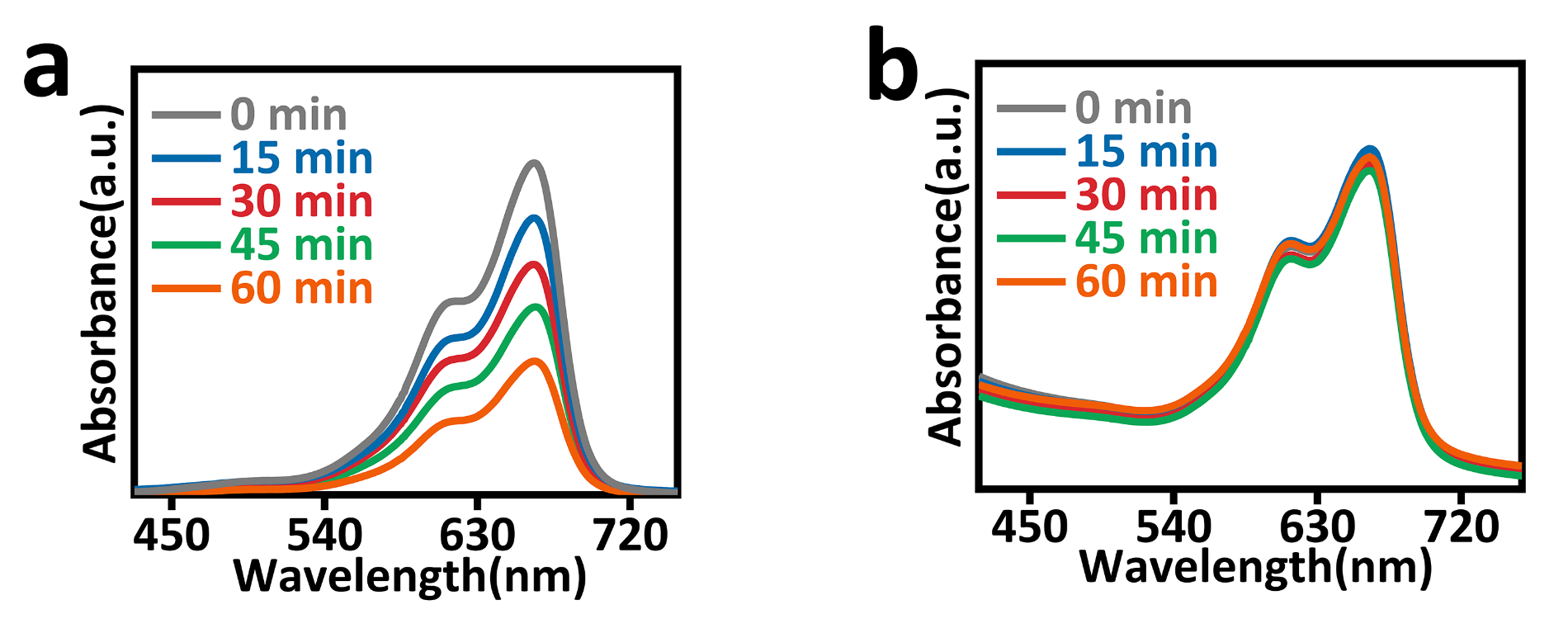


Fig. S10. UV-absorbance spectra of methylene blue (MB) degration by •OH. MB incubated with [H_2_O_2_] = 10 mM and a) [BBC] = 88 μg/mL, b) [BB] = 91 μg/mL at 37 °C.


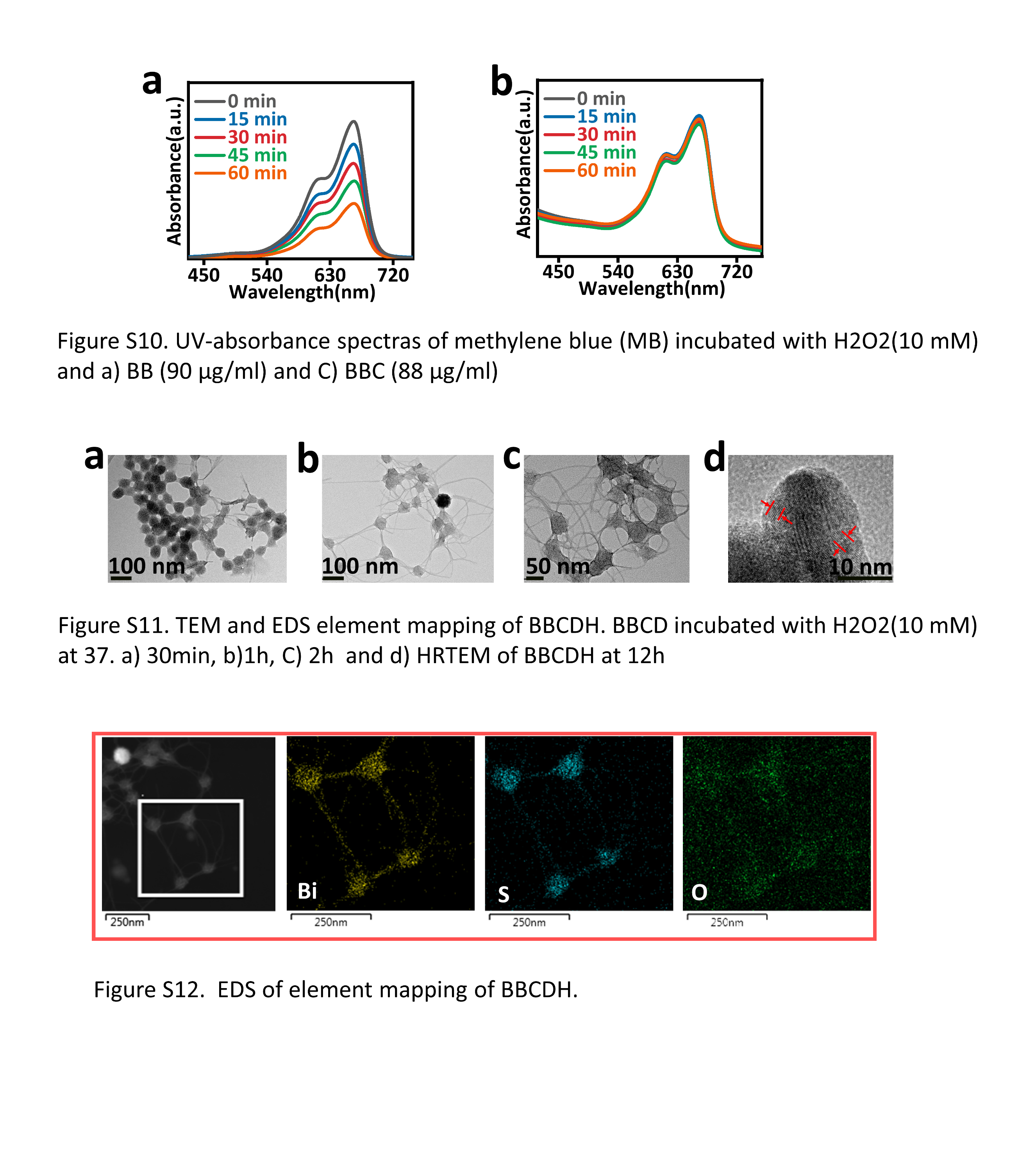


Fig. S11. TEM images of BBCD incubation with H_2_O_2_. BBCD incubated with [H_2_O_2_] = 10 mM at 37 °C. a) 30 min, b)1 h, C) 2 h and d) HRTEM image of BBCD_H_ at 12 h.


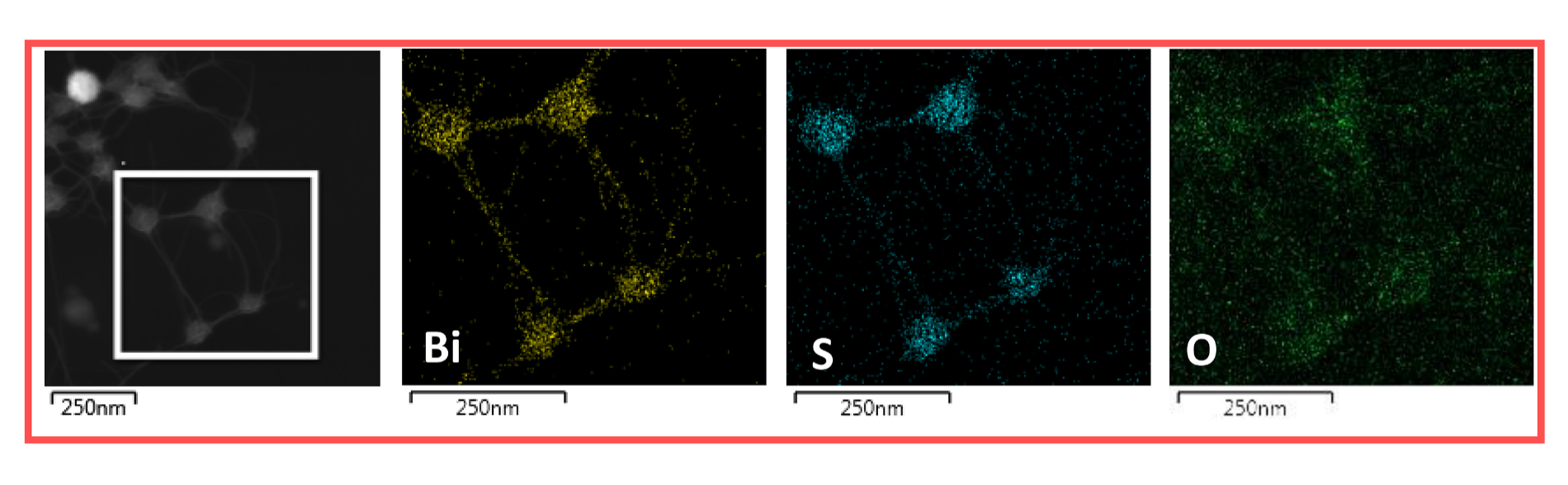


Fig. S12. EDS mapping of elements in BBCD_H_. BBCD incubated with [H_2_O_2_] = 10 mM at 37 °C for 12 h.


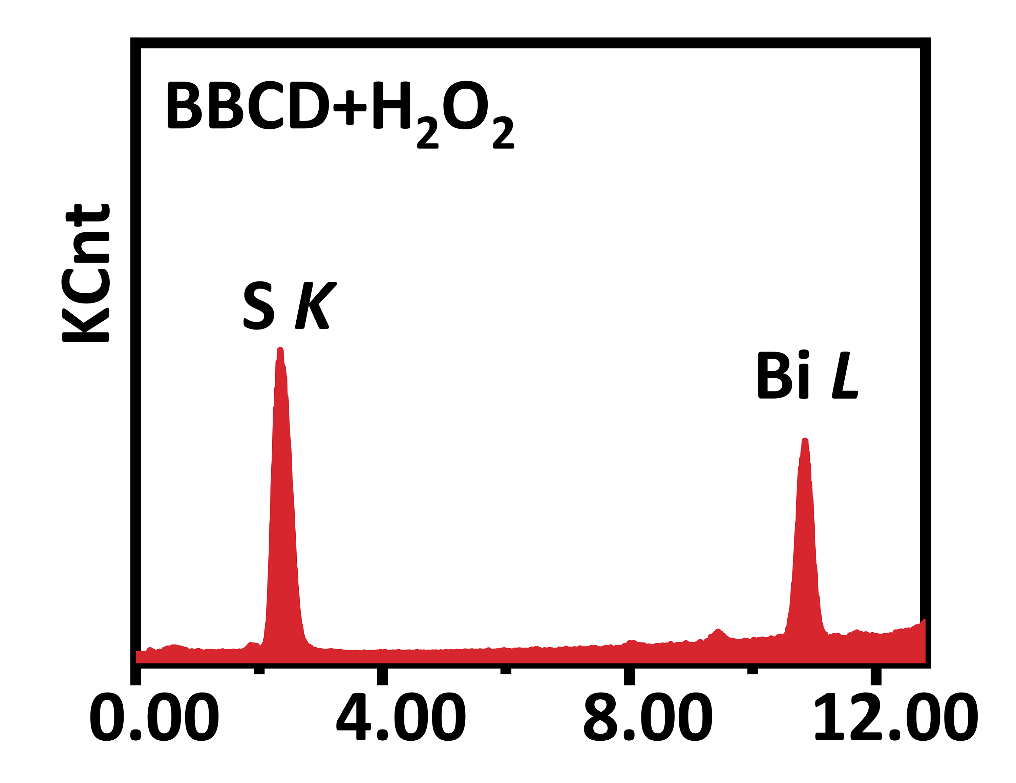


Fig. S13. XRF data of BBCD_H_. BBCD incubated with [H_2_O_2_] = 10 mM at 37 °C for 12 h.





Fig. S14. XRD data of BBCD_H_. BBCD incubated with [H_2_O_2_] = 10 mM at 37 °C for 12 h.


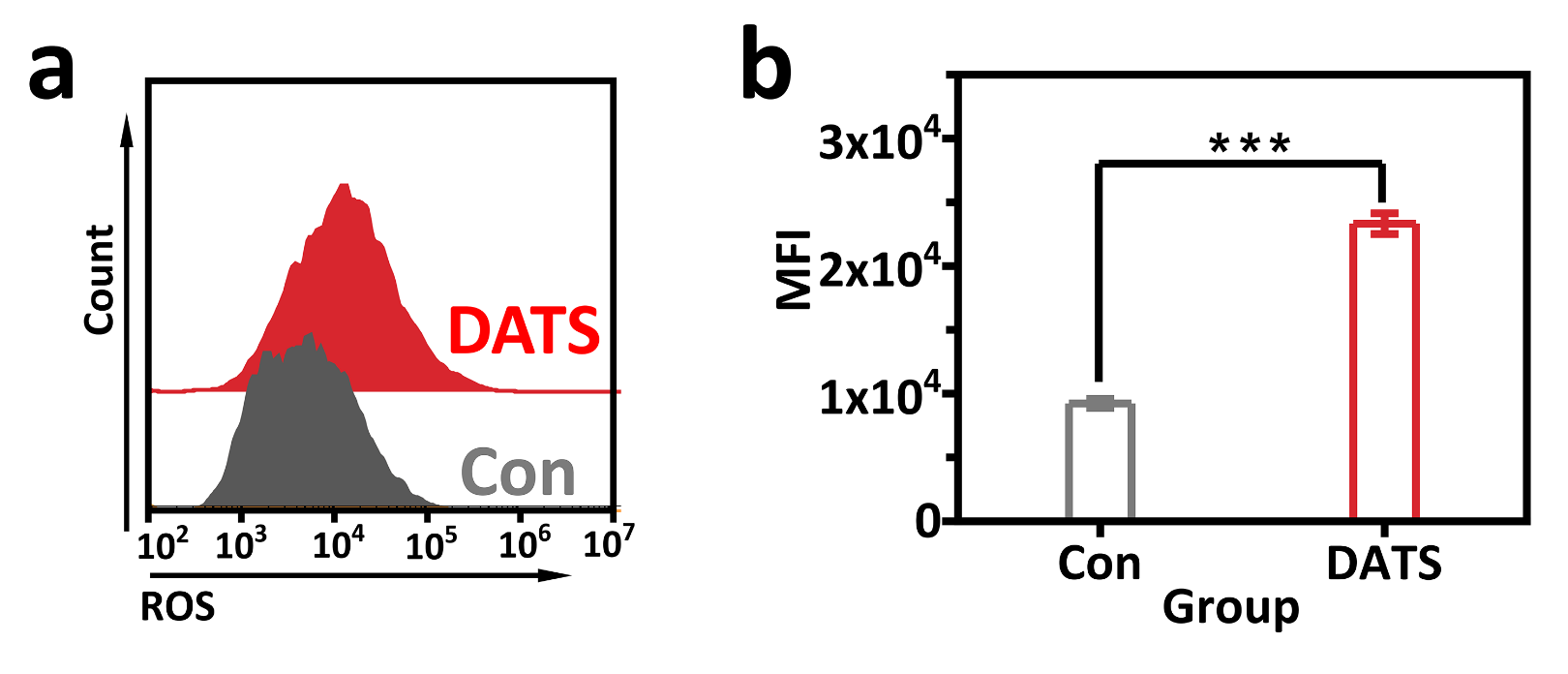


Fig. S15. Flow cytometry analysis for ROS in 4T1 cells. 4T1 cells incubated with [DATS] = 10 μg/mL for 12h.


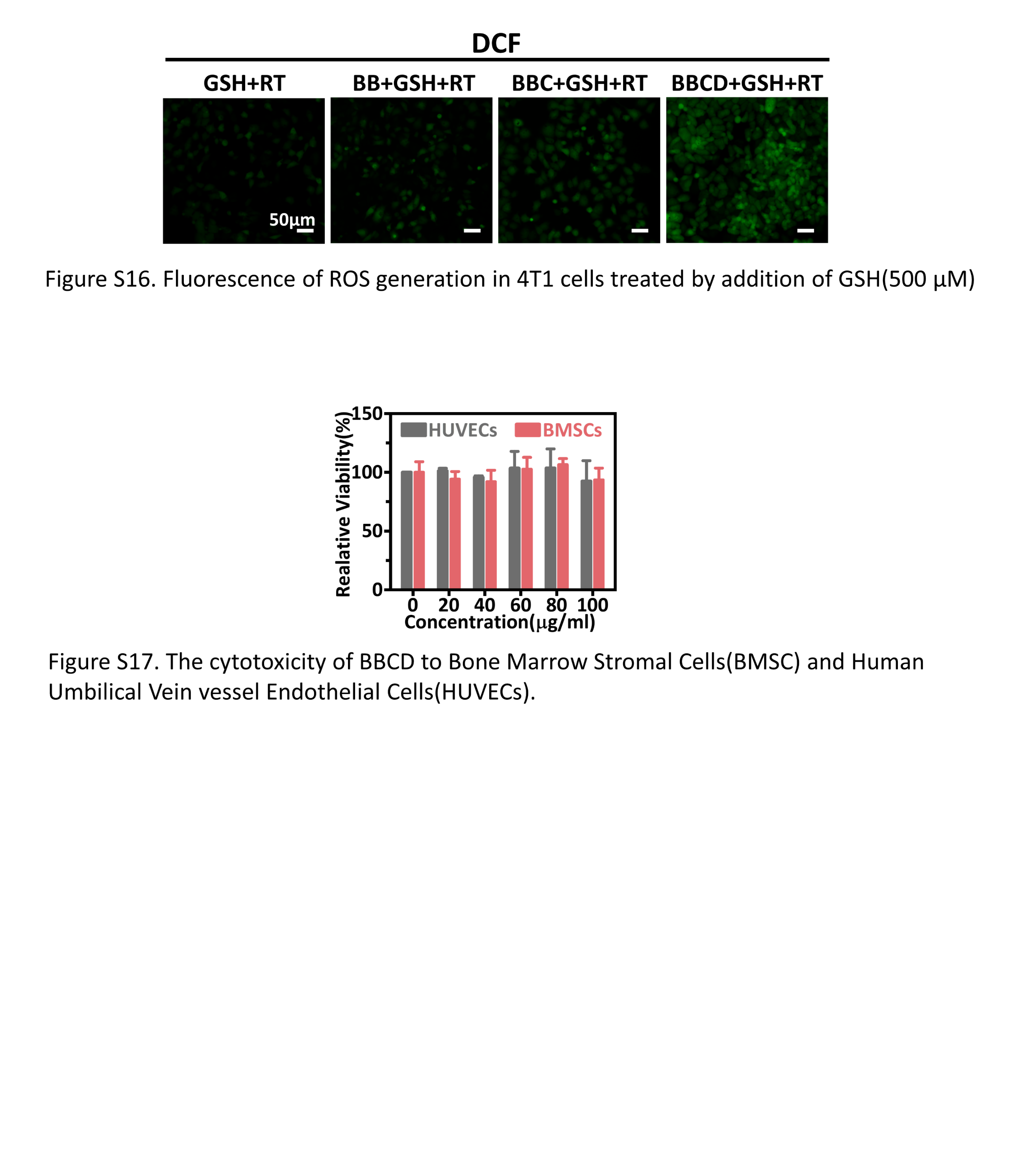


Fig. S16. Fluorescence images of ROS in treated 4T1 cells. [GSH] = 500 mΜ, [BB] = 91 µg/mL, [BBC] = 88 µg/mL, [BBCD] = 100 µg/mL, X-ray irradiation of 6Gy.


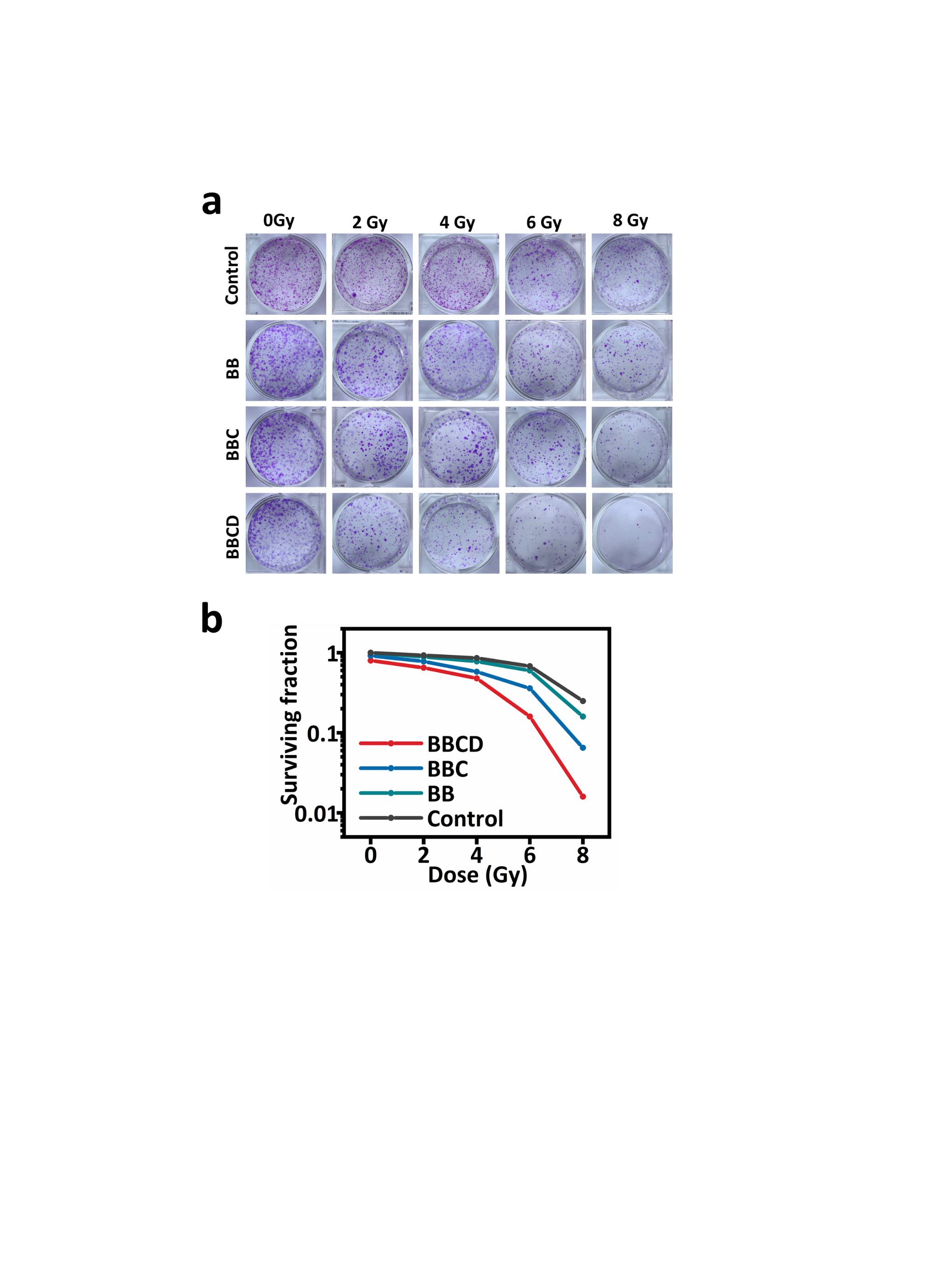


Fig S17. a, b) Colony formation assay of 4T1 cells. The 4T1 cells were pretreated with BBCD (100 µg/mL), BBC (88 µg/mL) or BB (91 µg/mL) for 12 h. 2000 pretreated cells per well were seeded into 6-well plates for 24 h, then treated with irradiation at the dose of 0 Gy, 2 Gy, 4Gy, 6 Gy and 8 Gy, respectively. After 7 days, the cells were fixed and stained with Giemsa.





Fig. S18. The cytotoxicity of BBCD to BMSC and HUVECs for 24h incubation.
